# Perceiving word structure in time: cross-linguistic evidence from English and Spanish

**DOI:** 10.1007/s11525-026-09469-2

**Published:** 2026-09-14

**Authors:** Clara Cohen, Matthew T. Carlson, Paola E. Dussias

**Affiliations:** 1 University of Glasgow, 12 University Gardens, Glasgow, G12 8QH UK; 2 The Pennsylvania State University, University Park, USA

**Keywords:** Speech perception, Phonetics, Phonology, Morphosyntax, Visual-world eye-tracking

## Abstract

Patterns of durational variation can offer rich phonetic cues to word structure, such as the number of syllables a word contains, or its morphological structure. In this study, we examine two types of durational cues: *direct phonetic cues*, which reflect phonological features, such as syllable count, and *ancillary morphophonetic cues*, which reflect phonological features triggered by morphological processes, such as suffixation. For direct phonetic cues, we focus on polysyllabic shortening, a pattern by which adding additional syllables to a stem (e.g., *cart* → *carton*) will shorten the duration of the initial syllable. For ancillary morphophonetic cues, we focus on segmental compression, a pattern by which adding a non-syllabic segment, such as a plural suffix, to a syllable coda (e.g., *cart* → *carts*) shortens segment durations in the syllable. In two visual world eye-tracking experiments, we ask whether listeners’ use of these cues in speech perception depends on (a) immediate morphosyntactic context of the word, (b) broader morphosyntactic features of the language, and (c) listeners’ bilingual language experience. Experiment 1, in English, reveals that English-speaking listeners use both types of cues, and further, that morphosyntactic context modulates listeners’ use of ancillary morphophonetic cues. Experiment 2 examines ancillary morphophonetic cues in Spanish, a language whose morphosyntax employs substantially more agreement than English. The results for Experiment 2 reveal that Spanish-speaking listeners, regardless of English experience, are more sensitive than English-speaking listeners to ancillary morphophonetic cues. These findings suggest that listeners’ use of ancillary morphophonetic cues reflects general perceptual processes, modulated by the role of these cues in their language’s grammar.

## Introduction

Listeners notice and exploit a wide variety of fine phonetic detail to support real-time speech perception. This is possible because phonetic detail reflects not random pronunciation bobbles, but linguistically structured variation. One linguistic domain that contributes to this structure is morphology. Durational variation in segments and morphemes offers a particularly rich source of cues to morphological structure. It can help distinguish words prefixed with *dis-* or *mis-*, such as *displease*, from pseudo-prefixed words that start with the same sequence, such as *display* (Smith et al., 2012). It distinguishes suffixal *-s* in English from stem-final *-s* (Plag et al., 2017; Schmitz, 2022); and it signals the presence of a following morpheme boundary (Sugahara & Turk, 2009). These patterns all directly reflect words’ morphological structure, and so offer direct morphophonetic cues to guide listeners’ real-time lexical access.

Another type of pattern, however, is not directly triggered by morphological structure, but rather arises as a consequence of the fact that adding morphological structure can trigger more general phonological processes related to word shapes, such as length and syllable complexity. We can distinguish two types of these phonologically-triggered phonetic cues. When they arise solely as a consequence of word shape, rather than morphological processes, they can be considered *direct phonetic cues*. Words with more syllables tend to undergo polysyllabic shortening, for example (Davis et al., 2002); and syllables with more complex codas can have shortened vowels (Munhall et al., 1992). However, when the addition of a syllable or the complexification of a coda is the result of a morphological process, such as suffixation, these cues can provide listeners with information about not only phonological structure, but also morphological structure. In this case, these details of pronunciation offer not just direct phonetic cues, but also *ancillary morphophonetic cues* to guide perception.

Both direct phonetic cues and ancillary morphophonetic cues allow listeners to infer properties of a word’s phonological structure — number of syllables, coda complexity — which in turn supports their identification of a word’s identity. However, ancillary morphophonetic cues allow listeners to infer not just word identity, but also morphological structure. When that morphological structure derives from inflectional morphology, ancillary morphophonetic cues can guide perception at levels other than lexical recognition, because inflectional morphology reflects properties of the syntactic structure of larger phrases. In languages with subject-verb agreement, the morphological form of the verb can connect subjects and predicates across large linear distances. In languages with number agreement, noun phrases can be produced discontinuously because the forms of the determiner and noun tie them together. The more pervasive the role of morphosyntactic agreement in a language’s grammar, the more potential there is for ancillary morphophonetic cues to affect listeners’ perceptual processing.

The goal of this paper is threefold. First we examine how listeners use direct phonetic cues and ancillary morphophonetic cues across different morphosyntactic contexts. Second, we ask whether the morphosyntax of a language affects a listener’s use of those cues. Specifically, we compare English noun phrases, whose morphosyntax is relatively impoverished, with Spanish, which enjoys robust, pervasive agreement between nouns, determiners, and adjectives. Finally, we ask how a listener’s language background influences their use of those cues.

### Direct and ancillary (morpho)phonetic cues

In order to understand what cues listeners might be drawing on, we will first briefly review evidence from production studies, which illustrate the difference between direct phonetic cues and ancillary morphophonetic cues.

#### Evidence from production

A classic example of a cue that can either be direct or ancillary is *polysyllabic shortening*. Polysyllabic shortening is the tendency of syllables in polysyllabic words to be shorter than segmentally equivalent syllables that form monosyllabic words. This pattern was first described in English in terms of morphological derivation: adding suffixes shortens a stem’s duration (Lehiste, 1972). Thus, words like *sleep* and *speed* are longer in duration than the first syllable of *sleepy* and *speedy*, which is in turn longer than the first syllable of *sleepiness* and *speedily*, despite sharing the identical segmental content. Crucially, polysyllabic shortening is not triggered by the addition of suffixes, per se, but by the addition of syllables. It may be therefore observed in word pairs that have no morphological relation to each other. For example, *Cap* is longer than the first syllable of *captain* (Davis et al., 2002) in the same way that *sleep* is longer than *sleepy*, even though *cap* and *captain* share a first syllable only by accident. In other words, the tendency of initial syllables to shorten when additional syllables are added is a phonological process, not a morphological process (White & Turk, 2010). When this polysyllabic shortening distinguishes different lexical items from each other by virtue of their syllable count, as in *cap* and *captain* (Davis et al., 2002), it is a direct phonetic cue. When it distinguishes an unsuffixed word form from a suffixed form of the same root, as in *clue* or *sleep* and *clueless* or *sleepy* (Blazej & Cohen-Goldberg, 2015; Lehiste, 1972), it is also an ancillary morphophonetic cue.

A similar situation can be observed in cases where syllable count remains constant. Even when affixation does not change the number of syllables of a word, it can still increase syllable complexity. Singular *knee*, for example, has no coda, while plural *knees*, bearing a suffix, does. Singular *rock* has a simple coda, while plural *rocks* has a complex one. In a phonological process known as *segmental compression*, syllables with complex onsets or codas tend to have shorter internal segmental durations – especially in the nucleus – than syllables with simple onsets and codas, or no codas at all (Katz, 2012; Munhall et al., 1992; Klatt, 1975; Crystal & House, 1990). Thus, just as affixation triggers polysyllabic shortening in a stem when it increases syllable count, it can also trigger segmental compression when the syllable count does not change. Plural suffixation in English can therefore also create ancillary morphophonetic cues.

The ability of a plural suffix to trigger segmental compression may be weaker than the ability of a syllabic suffix to trigger polysyllabic shortening. Adding a coda to an open syllable significantly shortens the duration of the vowel (Katz, 2012), but making a simple coda complex is not quite as reliable a trigger. Munhall et al. (1992) found shortening in vowel nuclei of less than 30 ms when an -s was added to syllables with voiceless codas of /k/ and /p/, and Shaiman (2001) found similar results with a small-scale articulatory study focusing on syllables ending with /p/. By contrast, Marin and Pouplier (2010) found no such pattern for voiceless plosive codas, and neither Marin and Pouplier (2010) nor Katz (2012) found evidence of vowel shortening when voiced *-z* was added to nasal codas. These findings are further complicated by the fact that stems tend to lengthen before morpheme boundaries (Sugahara & Turk, 2009), which means that a suffix may well be an even weaker trigger of segmental compression than non-morphemic coda consonants.

The studies on segmental compression cited above were all carefully controlled laboratory studies with small numbers of speakers. Munhall et al. (1992) had only three speakers, and the largest study used only seven (Marin & Pouplier, 2010). The stimuli were nonword syllables produced in simple phrases in all studies but that of Marin and Pouplier (2010), who used real words. Their variable findings may therefore simply reflect a lack of power to detect a small effect. Cohen and Carlson (2024) addressed this directly in a much larger corpus study of spontaneous American English. Rather than focusing solely on the vowel nucleus, they examined noun and verb stems as a whole, and observed that durations were reduced when a suffixal *-s* was added.

#### Evidence from perception

Listeners are able to draw on both direct phonetic cues and ancillary morphophonetic cues to guide their real-time speech perception. A substantial amount of research on polysyllabic shortening in English and Dutch has shown that listeners are better at processing single-syllable words when their stems are lengthened compared to shortened, and likewise better at processing polysyllabic words when their stems are shortened, compared to lengthened. This applies similarly, both when the polysyllabic option is related to the single-syllable form through mere accidental overlap in the initial syllable (Salverda et al., 2003; Shatzman & McQueen, 2006; Cohen, 2024; Davis et al., 2002), or through morphological suffixation (Blazej & Cohen-Goldberg, 2015; Kemps et al., 2005a,b).

Recent work has suggested that listeners can likewise make use of segmental compression as a cue to guide perception. Cohen (2024) conducted an experiment to examine how ancillary morphophonetic cues affect real-time sentence comprehension in English. In a visual-world eye-tracking study, she asked listeners to distinguish singular target nouns from plural competitors, embedded in sentences like *My view was blocked by the* *cart* *in front of the door*. Importantly, the duration of the noun stems was manipulated, so that singular nouns appeared in one of two conditions, ‘mismatch’ or ‘match’. In the mismatch condition, singular noun stems were shortened, in opposition to the ancillary morphophonetic pattern that produces lengthening in an unsuffixed word. In the match condition, singular noun stems were lengthened, consistent with that ancillary morphophonetic pattern. As predicted, listeners were better at identifying target nouns in the match condition than in the mismatch condition.

Cohen (2024) further reported that this effect was larger when the nouns followed an agreeing determiner, such as *this cart* or *that cart*, than when they followed a determiner with no morphosyntactic information: *the cart*. She proposes that the presence of an early morphosyntactic cue to the following noun’s number allowed listeners to predict the form of the noun — suffixed vs. unsuffixed — and thus develop expectations about the phonetic realisation of that noun. A singular-marked determiner indicated that an unsuffixed singular noun would follow, alerting listeners to expect a slightly lengthened noun-stem. A plural-marked determiner indicated the reverse. These determiners thus allowed listeners to begin building predictions about the form of the noun even before its acoustic onset. When a lengthened singular noun confirmed those predictions, speech perception was facilitated. When a shortened singular noun appeared, however, it impeded perception, both because the shortened form of the stem itself contradicted the prediction, and then again because the following suffix that would have triggered the observed shortening failed to appear. With non-agreeing determiners, on the other hand, listeners could not predict the expected form of the noun, which meant they neither benefited when those predictions were confirmed, nor suffered when the predictions were contradicted. In this way, the match effect was larger after agreeing determiners than non-agreeing determiners; and in this way, listeners’ real-time use of ancillary morphophonetic cues was modulated by morphosyntactic context.

The current study builds on these findings, by asking how generally these patterns are used across languages, and whether listeners’ use of them depends upon their language backgrounds. Before we can do that, however, we must first replicate these findings, to ensure that these results – in particular the modulating effect of morphosyntactic context on listeners’ sensitivity to segmental compression – are robust enough to build upon. This brings us to our first research question.

**RQ1:** Do listeners draw on direct phonetic cues and ancillary morphophonetic cues when perceiving English noun phrases, and does morphosyntactic context affect that process? In other words, can we replicate Cohen (2024)’s findings?

If these findings replicate, we will be in a position next to compare listeners in Spanish and English, to determine how processing of ancillary morphophonetic cues varies across languages with distinct patterns of morphosyntactic agreement. Whereas English has minimal agreement morphology, Spanish agreement is pervasive. If Spanish, like English, makes use of segmental compression as an ancillary morphophonetic cue, then the role of that cue in directing listeners’ sentence processing may differ from English, because the structure of Spanish morphosyntax is so different.

### Ancillary morphophonetic cues in Spanish

#### Evidence for segmental compression in production

Before we can consider how, precisely, segmental compression may vary across Spanish and English as an ancillary morphophonetic cue, it is first worth considering the evidence for whether such a cue exists at all in Spanish. Does adding a coda suffix in Spanish shorten a syllable? Since Spanish dislikes complex syllable codas, we will consider the simplest case first, that of adding a simple coda to a previously open syllable. In English, it is generally accepted that adding a coda to an open syllable will shorten what came before (Katz, 2012). In Spanish, the evidence is less consistent. In a small-scale reading study using texts from periodicals, Marín Gálvez (1994) found no difference in vowel duration between open and closed syllables. In a slightly larger study, Aldrich and Simonet (2019) asked twelve Spanish-speaking participants to read a list of nonwords embedded in a default phrase, and measured syllable nucleus duration across a variety of onset and coda structures. They observed that syllable nuclei shortened as onset complexity increased, but the same was not true for codas.

However, Marchini and Ramsammy (2022a) report observations that contradict Aldrich and Simonet (2019)’s claims: In Altiplateau Mexican Spanish and also Southern Chilean Spanish, coda consonants did trigger vowel shortening, with dialect-dependent nuances structuring the role of onset complexity and stress. Since Aldrich and Simonet (2019) do not give details about the specific dialects spoken by their participants, it is not clear whether these studies are genuinely at odds about the role of coda-triggered vowel shortening in Spanish, or whether they simply reflect the fact that different dialects of Spanish differ in their speech timing.

It is therefore unclear whether there is widespread segmental compression in Spanish, and consequently, it is unclear whether such cues are available to Spanish listeners for use during speech perception. This brings us to our second RQ:

**RQ2:** Do Spanish-speaking listeners use segmental compression in noun stems to perceptually distinguish unsuffixed singular nouns from suffixed plural nouns?

#### Segmental compression and morphosyntax

If Spanish-speaking listeners do use segmental compression as a perceptual cue, how might morphosyntactic context influence its use? Cohen (2024) observed that this cue has a larger effect (in English) following an agreeing determiner than following a non-agreeing determiner. This context-dependent use of an ancillary morphophonetic cue is particularly interesting when applied to Spanish, because Spanish uses substantially more morphosyntax to support number distinctions in nouns than English. English noun phrases can contain agreeing determiners, such as demonstratives (e.g., *these rocks*, *that chair*), but they just as often contain the non-agreeing determiner *the*. This means that an English-speaking listener will often lack early cues about the number of an upcoming noun. They will not know whether the noun is likely to be plural, with a more compressed pronunciation due to the additional coda consonant; or singular, with an uncompressed, lengthened noun stem. By contrast, Spanish determiners always agree in number with the following nouns, regardless of the identity of the determiner. Therefore, if a particular dialect of Spanish contains segmental compression, this ancillary morphophonetic cue will be strongly supported by the overt morphosyntactic agreement that nearly always precedes nouns.

Will this morphosyntactic support affect how Spanish listeners draw on segmental compression in real-time perception? On the one hand, the reliability of Spanish agreement might render segmental compression redundant as a perceptual cue, and so listeners may learn to disregard it. Evidence in favour of this learning mechanism can be found in Clayards et al. (2021)’s work on English. They asked whether listeners could predictively draw on direct morphophonetic cues which distinguished prefixed words like *discolour* from pseudo-prefixed words, like *discover*. They found that listeners could use such cues, but over the course of an experiment, the effect weakened. The authors attribute this change in effect size to rapid learning. The target words could be disambiguated effectively simply by waiting, and so listeners learned they did not need to attend to such cues. Spanish morphosyntax is so reliable a cue to noun number that it renders segmental compression maximally redundant. Hence, Spanish listeners may not attend to segmental compression, even if it is present in the language.

However, the materials used by Clayards et al. (2021) were evenly split between stimuli that matched and stimuli that did not match expected phonetic realizations. In other words, what participants may have learned was not that the phonetic cue was redundant, but that it was unreliable. By contrast, Cohen (2024) discusses a post-hoc analysis in the supplemental materials that finds a *strengthening* of the effect of her phonetic manipulation over the course of the experiment. She attributes this to the greater reliability of the target cue in her experimental materials: Fully 75% of the trials in Cohen (2024) reinforced the listener’s expectations that polysyllabic shortening and segmental compression signal additional syllables or suffixes after the noun root. Over the course of the experiment, the listeners could have learned that it was more reliable than not, and so began to use it more. By this reasoning, Spanish morphosyntax is so reliable a cue to noun number that it enables listeners to predict universally whether a following noun will be suffixed, and hence subject to segmental compression. The cue reliability would not teach listeners that the cue is redundant and to be disregarded, but rather that it is predictable and thus valid as a perceptual aid.

This brings us to our third research question:

**RQ3:** Do Spanish listeners differ from English listeners in their perceptual sensitivity to segmental compression in noun stems across different morphosyntactic contexts?

### The role of language experience

Even if Spanish listeners have little exposure to segmental compression in their own language, that does not mean they cannot learn about those patterns in other languages. An example of this can be found in Spanish-speaking listeners’ perception of word-initial consonant clusters beginning in [s], as in, e.g., *sports*. In English such clusters are common. In Spanish, by contrast, such clusters are phonotactically illegal, and loan-words containing such clusters are repaired with a prothetic [e] vowel. Carlson et al. (2016) report that monolingual Spanish listeners have difficulty identifying the presence or absence of very short vowels in word-initial position before consonant clusters, and instead tend to default to reporting hearing [e] – the only short vowel that is used to repair such illegal clusters. However, Spanish-English bilinguals perform better at distinguishing short vowels in that position. The authors attribute this finding to bilinguals’ exposure to English, which has enabled them to learn to attend to such distinctions. Carlson (2018) further reports that such perceptual changes can be detected in bilinguals who learned English at older ages than in Carlson et al. (2016), and that these changes are modulated by the degree of immersion in an English-speaking environment.

Carlson and colleagues’ stimuli consisted of isolated pseudo-words, rather than real words in sentence contexts, and so could not directly test listeners’ use of Spanish in perception. Nevertheless, the findings demonstrate that bilingual listeners are capable of learning L2-specific perceptual processing strategies. If listeners can directly import such strategies into their processing of their L1, then we can make predictions about the effects of English exposure on Spanish listeners’ sensitivity to segmental compression. Even if Spanish itself does not use segmental compression as an ancillary morphophonetic cue to signal the presence of a suffix, experience with a language which does use such cues may transfer into Spanish perceptual strategies. If so, we can predict that proficient Spanish-English bilinguals would show greater sensitivity to these cues than listeners with less experience in English.

There is a second reason to expect proficient bilinguals to show greater sensitivity to segmental compression. Although the so-called ‘bilingual advantage’ in language processing is hotly debated (see Bialystok et al. (2012) for a review of evidence in support, and Paap (2023) for the opposing view), there is also evidence that multilinguals have an advantage over monolinguals in phonetic and phonological processing. Antoniou et al. (2014), for example, found that bilinguals performed better than monolinguals at learning certain phonological contrasts. Tremblay and Sabourin (2012) observed that, not only did bilinguals outperform monolinguals, but multilinguals outperformed bilinguals. Spinu et al. (2018) also found a bilingual advantage in learning the sound pattern of a novel dialect, and proposed that it might arise from enhanced subcortical encoding of sound in bilinguals, relative to monolinguals (Krizman et al., 2012). Zhao et al. (2023) found that this effect is gradient, and affects native language listening as well as learning. In an oddball detection task using Mandarin stimuli, Mandarin-English bilinguals’ English proficiency predicted their performance, with higher English proficiency corresponding to faster RT. If bilinguals are at an advantage in learning and detecting sound categories, they may also have an advantage in learning and detecting sound patterns. For this reason, higher proficiency bilinguals may show a greater use of segmental compression in real time speech perception, compared to listeners with less multilingual experience.

Thus, whether through transfer, or benefits of bilingualism more generally, we can now articulate our fourth RQ:

**RQ4:** Do proficient Spanish-English bilinguals show greater sensitivity to segmental compression than listeners with less English experience?

### The present study

#### Overview

The present study replicates and builds upon Cohen (2024). In two experiments, English-speaking listeners (Exp 1) and Spanish-speaking listeners (Exp 2) were presented with audio recordings of sentences while viewing visual displays containing labelled pictures of four nouns. They were instructed to select the noun named in the sentence that they heard. Critical targets were always singular nouns (e.g., English *cart*, Spanish *coco*, ‘coconut’). These target pictures were always accompanied by one of two types of competitors. First, there were plural nouns (English *carts*, Spanish *cocos*, ‘coconuts’); second, there were what we shall call *carriers*, following Salverda et al. (2003): polysyllabic nouns that contained the singular noun embedded within it (English *carton*, Spanish *cocodrilo*, ‘crocodile’). This design of visual display ensured that exploiting segmental compression cues in the auditory signal could help participants distinguish singular targets from plural competitors, while exploiting polysyllabic shortening cues could distinguish singular targets from carrier competitors.

In Experiment 1, we examine how English-speaking listeners draw on both segmental compression and polysyllabic shortening, in a close replication of Cohen (2024). We differ from that study in two ways. First, we use slightly more experimental items, because the dialect of General American English used here maintains fewer distinctions than Scottish English. As a result, singular/carrier pairs like *bear/berry* and *tool/tulip* were possible here, while they were not possible in Scotland. Second, Cohen (2024) treated the magnitude of segmental compression cues and polysyllabic shortening cues as equivalent in the stimulus design. We distinguish between them, as detailed below in Sect. 2.1.4, in order to better reflect the patterns observable in naturalistic speech.

In Experiment 2, however, with Spanish-speaking listeners, we focus specifically on segmental compression. This is for two reasons. First, segmental compression is the only cue with morphosyntactic significance in this experiment: It can indicate an upcoming plural suffix *-s*, and so distinguish a singular target from a plural competitor. By contrast, polysyllabic shortening serves here only to distinguish singular targets from carrier competitors. Carriers have more syllables than the singular target, but bear no morphological relation. Thus, examining the role of polysyllabic shortening in Spanish would not shed any light on the possibility of a language’s morphosyntactic structure affecting listeners’ attention to ancillary morphophonetic cues. Second, we disregard the singular/carrier comparison because polysyllabic shortening in Spanish is badly conflated with stress position. Spanish tends to assign stress from the end of the word, usually in final position for consonant-final words; penultimate position for vowel-final words; and in antepenultimate position under certain (debated) phonological conditions (Fuchs, 2018). Our Spanish singulars were all two-syllable, vowel-final words, the simplest structure that ensures that a plural *-s* suffix can be added without increasing the syllable count (*coco* → *cocos*). For this reason, our singular nouns almost all had initial stress position. As more syllables were added in the carrier forms, these stress locations typically shifted rightward – e.g., *véna*, ‘vein’ → *venádo*, ‘deer’.^1^ Spanish uses duration as a primary, consistent cue to stress location (Ortega-Llebaria & Prieto, 2011), so shifts in the position of the stressed syllable mean that durational variation in the stem of the noun is confounded as a cue: it could serve either as a cue to stress position, or as a cue to polysyllabic shortening. Thus, Experiment 2 focuses only on the listeners’ use of segmental compression as a cue to plural suffixation.

In the audio stimuli for both experiments, the duration of the singular target noun was manipulated so that it was either shortened or lengthened. Recall that a shortened stem can be interpreted as the result of polysyllabic shortening or segmental compression. It indicates that the noun will likely end with additional syllables or a plural suffix. By contrast, a lengthened stem signals that additional syllables or suffixes are unlikely. Thus, in trials where the singular noun stem was lengthened in the auditory signal, listeners had access to cues that could aid in distinguishing target nouns from competitors. In trials where the stem was shortened, by contrast, the auditory signal contained cues that would mislead listeners.

Target nouns were presented in one of two contexts. In the Agreeing context, they were preceded by an agreeing determiner, as in (1a) and (2a). In the Non-Agreeing context, they were used in a metalinguistic mention context,^2^ as in (1b) and (2b).

**Figure:**
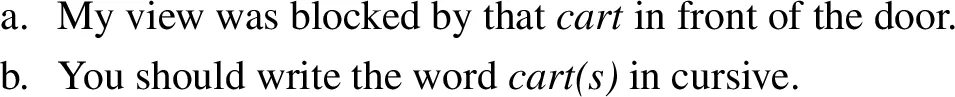
**Figure:**
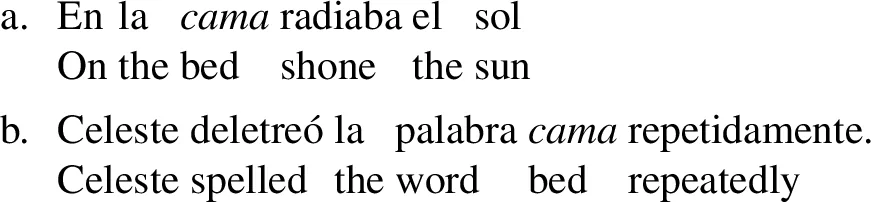

As participants viewed the displays, their gazes to the target and competitor pictures were tracked, to provide real-time information on their interpretation of the durational manipulation in the acoustic signal.

#### Predictions

Our prediction for Research Question 1 (RQ1) is straightforward: We expect that Experiment 1 should replicate the findings of Cohen (2024): English-speaking listeners should more easily distinguish singular target nouns from plurals and carriers when the nouns are lengthened, compared to when they are shortened. This pattern, which we shall call henceforth the *Match effect*, should differ across contexts. When participants must distinguish singular nouns from plural competitors, the Match effect should be exaggerated after agreeing determiners (e.g., 1a), compared to contexts without morphosyntactic agreement (1b).

RQ2 can be answered with the results of Experiment 2. If Spanish listeners use segmental compression as an ancillary morphophonetic cue to distinguish singular target nouns from plural competitors, then their results should be similar to the results in Experiment 1.

For RQ 3, if morphosyntactic agreement aids listeners in learning to use segmental compression, then Spanish listeners should show stronger sensitivity than English listeners. By contrast, if reliable morphosyntactic agreement in Spanish renders ancillary morphophonetic cues redundant, the Spanish listeners should show less sensitivity to segmental compression than English listeners.

For RQ 4, if exposure to a language with robust patterns of segmental compression transfers over into perceptual strategies in Spanish, or if bilingualism generally aids listeners in detecting durational cues to word structure, then higher proficiency in English should correspond to a larger Match effect in Spanish.

## Experiment 1

The goal of Experiment 1 is, first, to employ a visual-world eye-tracking paradigm to replicate the findings of Cohen (2024), and, second, to provide a baseline for comparison with Spanish. We examine English-speaking listeners’ sensitivity to polysyllabic shortening as a direct phonetic cue in order to directly replicate Cohen’s design, but the key focus here will be on listeners’ sensitivity to segmental compression as an ancillary morphophonetic cue across different morphosyntactic contexts. It is this latter effect that we will later compare with the Spanish results from Experiment 2.

### Methods

#### Participants

Thirty-two participants (26 female, 6 male, mean age = 19.5, SD = 2.9) were recruited from the Pennsylvania State University psychology subject pool. All were self-reported native English speakers, with minimal experience in Spanish (average of 1.9 years of experience), and a smattering of experience in other languages (e.g., Russian, Mandarin Chinese, Tagalog, Italian, French, German). Data from two subjects was discarded, one because abnormally flat corneas prevented an accurate read on the corneal reflection, and a second because of a self-reported hearing impairment. Participants were compensated with course credit or $15.

#### Materials

Stimuli were built around a set of 91 stems, which appeared in one of three forms: a singular (e.g., *cart*), a plural (*carts*), and a carrier (*carton*). The singular was always a one-syllable English word ending in a non-sibilant, which ensured that the plural would also be one syllable. The carrier was two syllables, and contained the singular form lexically embedded as its initial syllable. In critical trials, the target was always the singular noun, while filler trials presented plurals and carriers as the target.

In all trials, target words were presented auditorily, embedded in a sentence. While listening to the sentence, participants viewed a display that contained four images, one of which matched the target word. Participants’ gazes were tracked as they listened to the sentence and selected the target image.

**Fig. 1:**
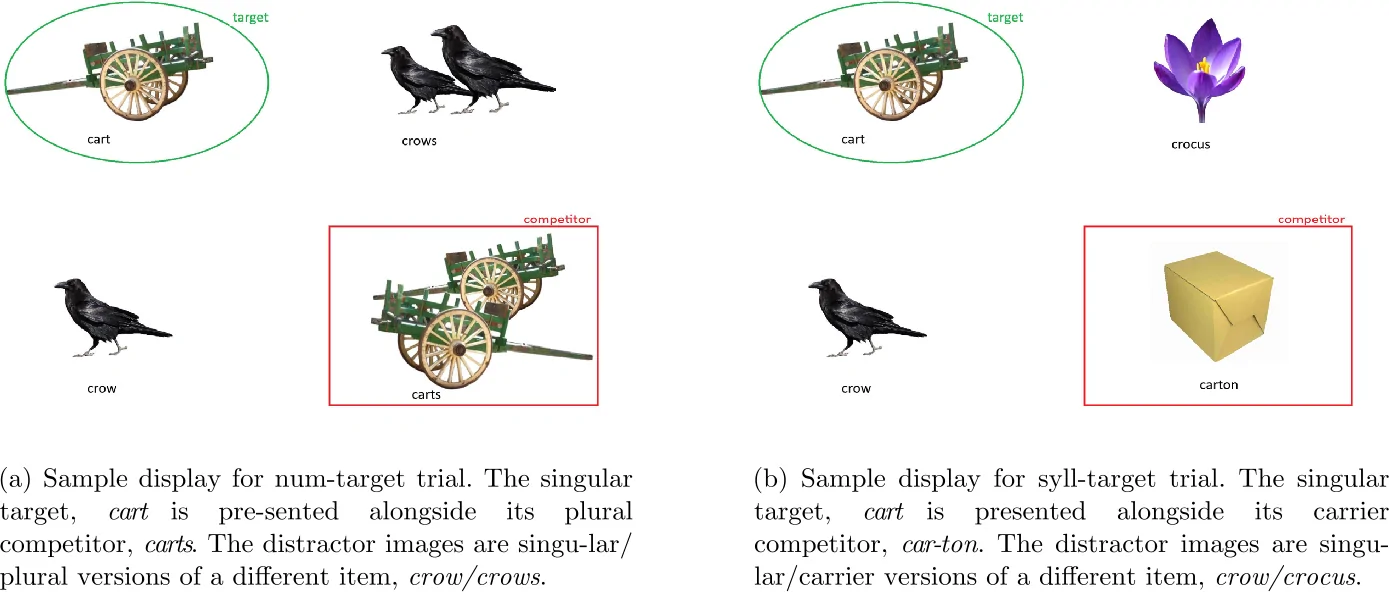
Sample displays for num-targets (a) and syll-targets (b). Note that the distractor images match the relationship between target and competitor

In critical trials, the target image — always the singular noun, e.g., *cart* — was presented alongside a competitor image, which could be either the plural counterpart of the target (*carts*), or the carrier counterpart (*carton*). We shall take a moment here to introduce some key terms. Singular targets that were presented alongside a plural competitor will henceforth be known as *num-targets*, because to identify them, participants must distinguish them from plural competitors, which differed in number. Singular targets that were presented alongside carrier competitors will be referred to as *syll-targets*, because participants needed to distinguish them from carrier competitors, which differed in syllable count. Examples of the num-target and syll-target visual displays are given in Fig. 1.

The surrounding sentence frame positioned each target word in one of two contexts. In the Agreeing context, the target was preceded by a determiner that signaled noun number, such as *that* or *those* (3a). In the Non-Agreeing context, the target appeared as a metalinguistic mention, with no determiner or cues to number (4a).

(3) Agreeing context:

**Figure:**
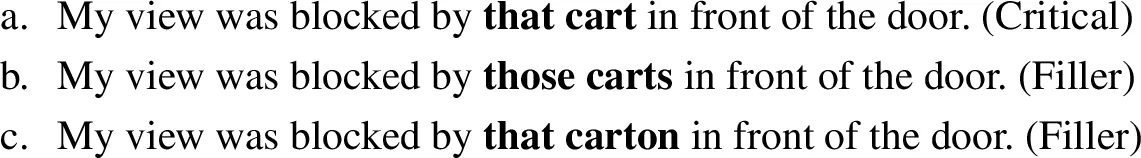
(4) Non-agreeing sentences:

**Figure:**
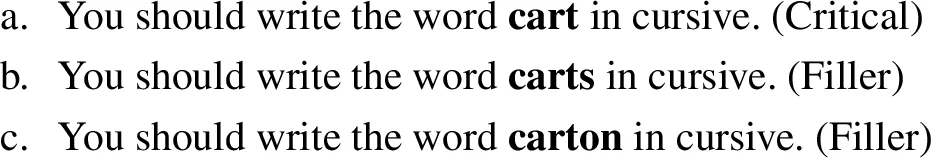

In both Agreeing and Non-Agreeing contexts, the word immediately following the singular target began with the same initial phones as the continuation of the carrier word. For example, in both the Agreeing sentence frames (3a) and the Non-agreeing sentence frames (4a), the word following *cart* is *in*. When pronounced in a General American accent, this reduces to something quite similar to the final syllable of *carton*. As a result, it is difficult for a listener to know whether they are hearing *carton* () or *cart in* () until later in the sentence. We included this ambiguity to maximize the period in which listeners would have to draw solely on durational cues, which were the experimental manipulation, to identify the target before later phonemic information became available. We were not able to include a similar manipulation for num-targets, because it was not possible to construct sentences in which every word following a num-target began with /s/ or /z/. We return to the question of suffix-voicing in the Discussion.

The full list of stimuli is included in our OSF archive for this paper: https://osf.io/u7y6z/overview?view_only=716487e43450451ab3d6db240ddf17f4.

#### Recording and stimulus check

The raw recordings were produced by a phonetically trained female native speaker of American English, who took pains to ensure that all sentences were pronounced with as similar an intonation as possible in the portion before the target word. They comprised a set of six sentences for each stem, crossing the three word forms (singular, plural, carrier) with the two sentence contexts (Agreeing, Non-Agreeing) to produce a set of raw recordings of the sort shown in (3-4).

**Fig. 2:**
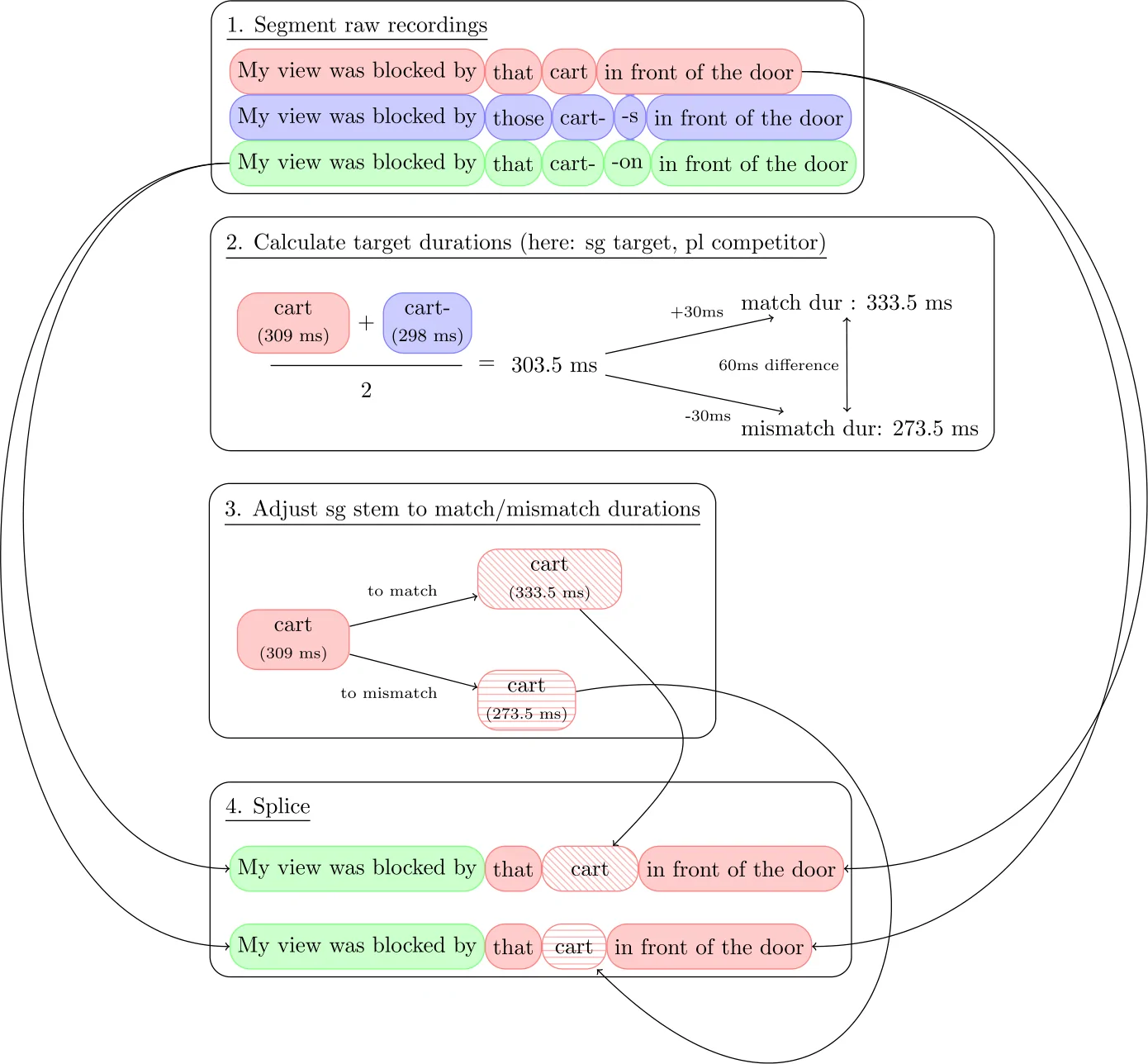
Algorithm for creating critical stimuli containing singular targets designed to be presented with a plural competitor. (1) Raw sentences are first segmented into preambles, determiners (if applicable), stems, and postambles. (2) The target durations for match and mismatch stems are then calculated; in this case, the target durations are set so that there is a 60 ms difference between the match and mismatch stems, with the match stems 30 ms longer than the average duration of the singular and plural raw durations, and the mismatch stems 30 ms shorter. (3) The extracted singular stem is adjusted to the target match and mismatch durations. (4) The sentences are spliced together, forming match and mismatch stimuli. In the scenario presented in this diagram, the carrier preamble is used, followed by the original determiner from the singular raw recording, followed by the adjusted stem, and finishing up with the original postamble from the singular raw recording

Acoustic manipulation proceeded as summarised in Fig. 2. The first step was to segment each of the raw recorded sentences into the following component elements: The preamble consisted of everything up to the onset of the determiner in Agreeing sentences, and everything up to the onset of the stem in the Non-agreeing sentences. The determiner (if applicable) followed, with the stem next, followed by the suffix (for plurals) or remaining syllables in the word (for carriers). The postamble consisted of everything after the offset of the word.

We examined the duration of the stems in our raw recordings, to see whether there was evidence for segmental compression and polysyllabic shortening. The results are shown in Table 1. Although stems ending in voiceless consonants showed reasonable evidence of both patterns, stems ending in voiced segments did not seem to show any evidence of segmental compression. However, these patterns were produced by a single speaker, phonetically trained, who took pains to make each sentence as comparable as possible to its counterparts with the other forms of a given noun stem. It is possible, therefore, that these raw recordings do not represent the patterns that listeners typically hear, and in fact minimize durational differences across word types. Accordingly, we decided to investigate further how listeners made use of the cues in the raw recordings.

**Table 1:** Mean stem durations (and standard deviations) in ms from the raw recordings of English stimuli, along with the durational difference from the singular counterpart

Context	Voiceless stems			Voiced stems		
	singular	plural	carrier	singular	plural	carrier
Agreeing	322 (42)	301 (41)	277(42)	328(51)	347(49)	243(44)
dur. dif. from sg.	0	−21 (34)	−45(28)	0	19(35)	−85(35)
Non-agreeing	384(43)	341(46)	302(43)	367(55)	375(54)	258(44)
dur. dif. from sg.	0	−43(32)	−82(32)	0	8(26)	−109 (39)

To this end, we ran a brief experiment, in which the raw stems of singular and plural target words were extracted from their surrounding context and presented to listeners. Listeners (n = 19) were presented with these recordings and asked to respond whether they thought these stems came from an originally singular word, with no suffix, or an originally plural word, with the *-s* suffix. Stimuli were split up into two lists, so that each listener heard a given stem twice, once from the Agreeing sentence and once from the Non-agreeing sentence, with one of those trials from an original singular and the other from an original plural. The responses (singular vs. plural) were analyzed with mixed effects logistic regression modeling in the lme4 package (version 1.1, Bates et al., 2015) in R (version 3.2.4, R Core Team, 2025). The model-building procedure used forward addition of predictors, with a log-likelihood ratio test to assess the improvement of fit to the model of each added predictor. The initial model included only log-transformed stem duration, with random effects comprising de-correlated intercepts and slopes for stem duration by subject and item. To this simple model were progressively added the original stem type (singular vs. plural), and whether the final segment of the stem was voiced or voiceless.^3^ Adding an interaction between voicing and stem duration did not improve model fit ($\chi ^{2}(1) = 1.27$, *p* = .259).

The final model, summarized in Table 2, indicates that listeners did draw upon duration in the raw stimuli as a cue to word type: The longer the duration, the more likely they were to respond singular. Duration did not explain everything, however: Over and above any effect of stem length, listeners were still more likely to respond singular when the stem came from an originally singular word. This could have been the result of coarticulatory effects: Singular stems were extracted from a context that did not necessarily include a following alveolar consonant, while all plurals did have the alveolar fricative originally following the stem, which could have yielded formant transitions alerting listeners to the missing [s] or [z] suffix. Finally, the voicing of the stem’s final segment had an effect beyond any effect of duration: Stems with voiced final segments were less likely to be classified as singular than those with voiceless final segments. Importantly, voicing did not interact with duration, indicating that listeners used stem duration similarly regardless of whether the final consonant was voiced or voiceless.

**Table 2:** Model summary for the results of the perception experiment on raw recordings

Term	Estimate	Std. Error	*z*	*p*
(Intercept)	4.073	0.441	9.242	<.001
log-transformed duration	2.696	0.355	7.585	<.001
word type = sg	0.325	0.079	4.143	<.001
voicing = voiced	−1.060	0.211	−5.033	<.001

The key result of this norming experiment is the significant effect of stem duration: Even taking into account properties such as whether the word has a voiced or voiceless final segment, and especially whether it originally had a suffix and hence coarticulatory cues as to the suffix’s presence, listeners still used duration to guide their classification. Longer stems were more likely to be classified as singular, and short stems were more likely to be classified as plural. Despite the lack of robust evidence of segmental compression in the raw recordings, listeners seem to expect it to some degree, and draw on this expectation when making their judgments.

#### Manipulation

After analysing the raw duration of the recordings, we manipulated the durations of the target words to systematically carry the relevant durational cues, as laid out in Fig. 2, steps 2-4. These manipulations produced two conditions: *Match* and *Mismatch*. To create the Match condition, we lengthened the target word’s duration, so that it lacked any polysyllabic shortening or segmental compression, consistent with the expected phonetic realization of a one-syllable, unsuffixed singular noun. To create the Mismatch condition, we shortened the target word’s duration, so that it carried evidence of either polysyllabic shortening or segmental compression. This shortening was designed to be misleading, since the target word would eventually reveal itself to be a single-syllable, unsuffixed singular noun after all, despite the shortening in the stem. The temporary confusion as listeners recover from their misled expectations is the source of the Match effect.

To determine the appropriate degree of shortening or lengthening necessary to produce the match manipulation on noun stems, we considered three factors: the durational patterns of our own raw recordings; the durational patterns reported in other literature; and the degree of durational manipulation that produced a perceptible difference, based on our own auditory judgment of our stimuli.

The raw recordings, summarized in Table 1, showed a maximum mean difference in stem duration of 109 ms between singular nouns and the first syllable of carriers. This is comparable in magnitude to reports from other studies of English polysyllabic shortening. Blazej and Cohen-Goldberg (2015) analysed their own raw recordings and report a mean difference of 81 ms between first-syllable duration of unsuffixed and suffixed words. Lehiste (1972) reports syllable durations for one-syllable words and their two-syllable suffixed counterparts which average out to a mean difference of 85 ms. We settled on a durational manipulation of 90 ms for our syll-targets.

By contrast, the durational differences between singulars and the stems of plurals were substantially smaller, with a maximum mean difference of 43 ms. This suggested to us that a smaller manipulation would be appropriate for the num-targets compared to syll-targets. However, auditory evaluation of manipulated stimuli with a durational difference of 40 ms did not strike us as sufficiently perceptible. Further, corpus work by Cohen and Carlson (2024) reports a durational reduction of between 90-100 ms when a suffixal -s is added to a noun stem, which would support a more extreme difference than 40 ms. Accordingly, we compromised between the numbers from the corpus study and the numbers from our own raw recording, and chose a target difference of 60 ms.

This decision to use a smaller Match-Mismatch difference for the num-targets (60 ms) compared to the syll-targets (80 ms) supports a secondary experimental goal. Cohen (2024) constructed the durational manipulation to be an equivalent 80 ms in both num-targets and syll-targets. The results revealed a larger Match effect in syll-targets than in num-targets. One possibility for this difference is that polysyllabic shortening is simply a more valuable cue than segmental compression for identifying words. However, another possibility is that polysyllabic shortening reduces duration more than segmental compression. Certainly the data from our raw recordings in Table 1 supports that hypothesis. Therefore, perhaps the smaller Match effect observed by Cohen (2024) reflects the fact that the acoustic manipulation in that experiment did not respect the differences between polysyllabic shortening and segmental compression. Listeners may not have been able to interpret the segmental compression effectively, because it sounded identical to polysyllabic shortening. In this experiment, therefore, we address this possibility in the construction of our Match and Mismatch stimuli, by building in a smaller durational difference for num-targets than syll-targets.

After determining the desired durational difference between the Match and Mismatch conditions, we adjusted the duration of all stems, using the overlap-add implementation of PSOLA in Praat (Boersma & Weenink, 2015). All targets had their durations adjusted, both for critical trials and filler trials. In a trial with a singular target and a plural competitor — i.e., for num-targets — we first averaged together the raw durations of the singular and plural recordings; to this we then added 30 ms to arrive at the desired Match, and subtracted 30 ms to arrive at the desired Mismatch duration (Fig. 2, step 2). In a trial with a singular target and carrier competitor — i.e., for syll-targets — we first averaged together the singular and carrier raw stem duration, and then added 45 ms to determine the desired Match duration, and subtracted 45 ms for the Mismatch duration. This process ensured that the duration difference between Match and Mismatch num-targets was 60 ms, and the difference between match and mismatch syll-targets was 90 ms (Fig. 2, step 3).

A final consideration was the possibility that the preamble might contain long-distance cues that would point the listener to expect either the target or its competitor. We therefore took the adjusted target word, and spliced it onto the preamble of the recording that contained neither it, nor its intended competitor. For example, a num-target *cart* would have a plural competitor *carts*, so we spliced the adjusted num-target onto the preamble from the recording containing the carrier, *carton*. A syll-target *cart* would have the carrier competitor *carton*, and so we spliced the adjusted syll-target onto the preamble from the recording containing the plural, *carts*. It was still possible that the preamble would contain cues pointing to the original word that was produced in the raw recording, but this splicing procedure ensured that any cues remaining in the preamble would at least point to a word that was not present on the screen (Fig. 2, step 4). It further ensured that the Match and Mismatch forms of each item appeared after an identical preamble, thus controlling for any possible effects of speech rate on listeners’ use of durational cues (Dilley & Pitt, 2010; Pitt et al., 2016).

Filler sentences contained plurals and carriers as their targets. All filler targets were also adjusted in duration and spliced onto a different preamble, to ensure that any artifacts of the durational adjustment were present throughout the experiment, rather than confined to critical stimuli. However, rather than creating Match and Mismatch versions of the filler targets, we used only the shortened durations. As a result, 75% of trials on each experimental list presented targets with durational cues that corresponded to their word structure. All filler targets were appropriately shortened, and half of the critical targets were appropriately lengthened. This design reinforced the fundamental experimental assumption that singular words are longer than plural or carrier competitors. In all filler trials and half the critical trials, the stimuli present a 60 ms difference between lengthened singular and shortened plural stem durations, and a 90 ms difference between lengthened singular and shortened carrier stem durations. Across each stimulus list, only 25% of the experimental stimuli contained durational cues that contradicted listeners’ expectations.

#### Design

For each of the 91 stems, eight critical sentences were created. Four of these focused on the role of polysyllabic shortening. These four sentences all contained a singular syll-target in one of four conditions, formed by crossing the factors of sentence Context (Agreeing/Non-Agreeing) with duration Match (Match/Mismatch). The other four sentences focused on the role of segmental compression. They all contained a singular num-target, again representing the four conditions formed by crossing sentence Context and duration Match. All eight sentences were rotated across eight experimental lists so that no critical target word was presented twice in the same list.

Each list contained an additional 91 fillers, which all contained as the target words the plural or carrier forms of the same 91 stems. In each list, the plural or carrier filler appeared in the opposite sentence context from its singular counterpart. For example, in list A the word *cart* appeared as a syll-target, with a carrier competitor, in a Non-agreeing sentence. The filler counterpart was therefore the plural *carts*, presented in an Agreeing sentence. In this way, each list contained two instances of each stem in different contexts. In one occurrence the stem appeared as a singular target, and in the other as whichever type of filler—plural or carrier—did not appear as the singular target’s competitor.

The visual world for each trial contained four images: the target, its competitor, and two distractors (see Fig. 1). In critical trials, the target was always singular, and its competitor could be either plural (for num-targets) or carrier (for syll-targets). In filler trials, the target was either plural or carrier, and its competitor could be either of the other two word types. Thus, if a filler target was a carrier, its competitor could be either plural or singular; and if a filler target was plural, its competitor could be either carrier or singular. In critical trials, distractor pictures were selected to mirror the relationship between target and competitor, whereas in filler trials, the relationship between target and competitor was mirrored in the distractors only half the time.

#### Procedure

The experimental procedure began with collecting informed consent from participants, after which they completed the visual-world eye-tracking experiment. Following the eye-tracking experiment, participants completed the LEAP-Q language history questionnaire (Marian et al., 2007), the MINT naming task (Gollan et al., 2012), a verbal fluency task, and a subset of the Michigan English Language Institute College Entrance Test (MELICET).

Eye-tracking data was collected from the right eye at a 1000 Hz sampling rate with an Eyelink 1000 camera; stimuli were presented using the Experiment Builder software, with audio stimuli playing binaurally over headphones at a comfortable volume. Participants were instructed that they would see four images appear on the screen, and hear a sentence mentioning one of the images. They were instructed to use the mouse to select the picture on the screen that was named in the sentence. Each experiment began with a nine-point calibration and validation, followed by four practice trials, followed by the 182 trials of the experiment itself. Every 45 trials, participants had a break, after which the equipment was recalibrated before resuming. Fillers and critical trials were randomly presented in each list, with the restriction that no particular combination of target type (singular, plural, carrier) and competitor type was presented more than twice in a row.

Each trial began with a fixation point on the screen. After participants fixated upon the point, four images appeared on the screen, each labeled underneath the image. Participants had four seconds to look freely at this screen to familiarize themselves with the pictures’ names and locations before the audio began to play. Participants could not select an image before the audio began to play, but they were free to click on an image before the audio was completed. If they selected the correct image, a green frame appeared around the selected image; if they selected the wrong image, a red frame appeared. Regardless of their selection, the entire audio file played to completion before the screen reset to the fixation cross. From start to finish, the entire procedure took about 1.5 hours.

#### Analysis

Gaze trace samples were binned in two stages into windows. This is because we wanted to include the target word itself in the interest period, but it varied in duration – both as a natural result of the different segmental content, but also as a consequence of the Match manipulation. For this reason, uniform bins would have made it complicated to anchor analyses to the offset of the target word. Therefore, we set the bin size for the period of the target word to be 1/5 of each target’s respective duration. The first five window bins could be as short as 31 ms or as long as 104 ms, but they all agreed that the end of window bin 5 marked the offset of the target word. As the results will show, all effects of interest emerged only after the offset of the target word. Following the offset of the target word, each window was set at 50 ms.

Gaze locations to the target and competitor were then counted up as a proportion of all readable samples within each window bin. Blinks and other tracking failures were thus excluded from the calculations, but saccades were included, as participants often saccaded from one location to another in the same image. Proportions of looks to target and looks to competitors were converted to empirical logits (elogs), following Barr (2008). We then calculated target advantage, by subtracting elog looks to competitor from elog looks to target. Elog target advantage was the dependent variable.

Visualisation of elog target advantage over time of the full dataset revealed that looks to target peaked around time window 19, which is 14 windows, or 700 ms, after target offset. Accordingly, we restricted our analysis to the interest period from target onset to 700 ms after target offset.

**Fig. 3:**
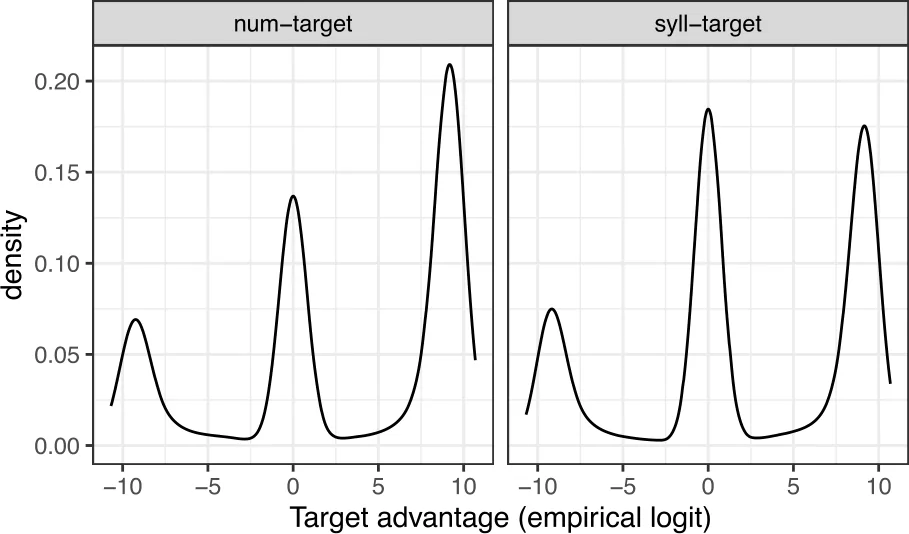
Density of elog target advantage for syll-targets (left) and num-targets (right), showing trimodal distribution of values. Left-hand density peaks reflect windows when the gaze fixates entirely on the competitor; middle density peaks reflect windows when the gaze is on neither target nor competitor; right-hand density peaks reflect windows when the gaze fixates entirely on the target

Elog target advantage of the gaze traces was analysed in the R programming environment (version 4.4.3, R Core Team, 2025) with empirical Bayesian non-parametric quantile regression modeling (QGAMs; Fasiolo et al., 2021; Baayen et al., 2022), an extension of generalized additive mixed modeling methods (GAMMs, see Sóskuthy, 2017; Wood, 2011, 2017). QGAMs analyse quantiles as the dependent variables, rather than means as GAMMs do. They have the advantage of being unconstrained by assumptions about the distribution of the dependent variable, which makes them particularly useful for eye-tracking data of this sort. As Fig. 3 shows, the distribution of elog target advantage was highly non-normal. This is because fixations tend to be longer than 50 ms, so even after binning, most window bins contained either entirely looks to target, entirely looks to competitor, or entirely looks elsewhere. As a result, the dependent variable followed a trimodal distribution. Increasing the window size might have minimized the trimodality of the distributions, but at a loss of granularity in the dynamic time course of gaze traces. For this reason, we analysed median elog target advantage as our dependent variable, using QGAMs rather than GAMMs.

The QGAM analysis used the package qgam for model building (Fasiolo et al., 2021, version 1.3.4,) and itsadug for model comparison (version 2.4.1 van Rij et al., 2022). QGAMs (and GAMMs) are similar to linear mixed effects models, except they include two different types of predictor variables. *Parametric terms* in QGAMs operate similarly to terms in a linear mixed effects model: Their coefficients capture the overall (median) height of the curve across the entire time period of interest (here, the first 19 time windows). The *smooth terms* capture the curve shapes of the gaze trajectories across that time period. Thus, if two gaze trajectories cross, such that one rises faster and peaks earlier than another before declining, the parametric terms might indicate little difference between the trajectories, as their overall median height is similar across the entire interest period. The smooth terms, however, would capture the fact that one trajectory reflects faster identification of the target than the other.

Target advantage for num-targets and syll-targets was analyzed in separate models, to prevent an unnecessary profusion of interactions between target type and other variables of interest. As none of our research questions relate to the possibility of differences between num-targets and syll-targets, we elected to keep the modeling procedure simpler and more interpretable. In both cases the model-building procedure was similar. Parametric terms for Context (Agreeing/Non-Agreeing) and Match (Match/Mismatch) were effects-coded, with Agreeing and Match set as 0.5, and Non-Agreeing and Mismatch set at −0.5. Thus the parametric effect of Context reflects the overall median advantage in elogs of Agreeing contexts over Non-agreeing contexts, and the parametric effect of Match reflects the overall median advantage in elogs of Match targets over Mismatch targets. Smooth terms were treatment-coded. The baseline smooth reflected the default level of relevant factors (Match, Agreeing context), and subsequent smooths show the difference in curve shape between the baseline curve and the curve associated with the other factor level. Higher Effective df values indicate greater differences in curve shapes.

All models began with the maximum structure, including interactions between Context and Match in both parametric and smooth terms, and random factor smooths by subject and item. These models were incrementally simplified to remove non-significant terms. Full code for the model-building procedure is available on an OSF archive: https://osf.io/u7y6z/overview?view_only=716487e43450451ab3d6db240ddf17f4.

### Results

#### Syll-targets

The final model for the syll-targets included parametric terms for Match and Context, and a smooth term for Match alone. The full model summary is given in Table 3.

**Table 3:** Model summary of target advantage for looks to singular syll-targets. Parametric intercept represents the overall median. The ‘baseline’ smooth represents the curve for Match, while the following term gives the difference smooth for the Mismatch curve

*Parametric terms*				
Term	Estimate	Std. Error	*z*	*p*
(Intercept)	2.48	0.23	10.70	<.001
Match	1.19	0.08	15.32	<.001
Context	−0.55	0.08	−7.04	<.001
*Smooth terms*				
Term	Effective df	Ref.df	$\chi ^{2}$	*p*
Baseline	5.83	7.18	31.91	<.001
Match=Mismatch	4.29	6.03	4.99	<.001
(by-subject smooths)	109.34	299.00	731.35	<.001
(by-item smooths)	364.51	909.00	2639.21	<.001

**Fig. 4:**
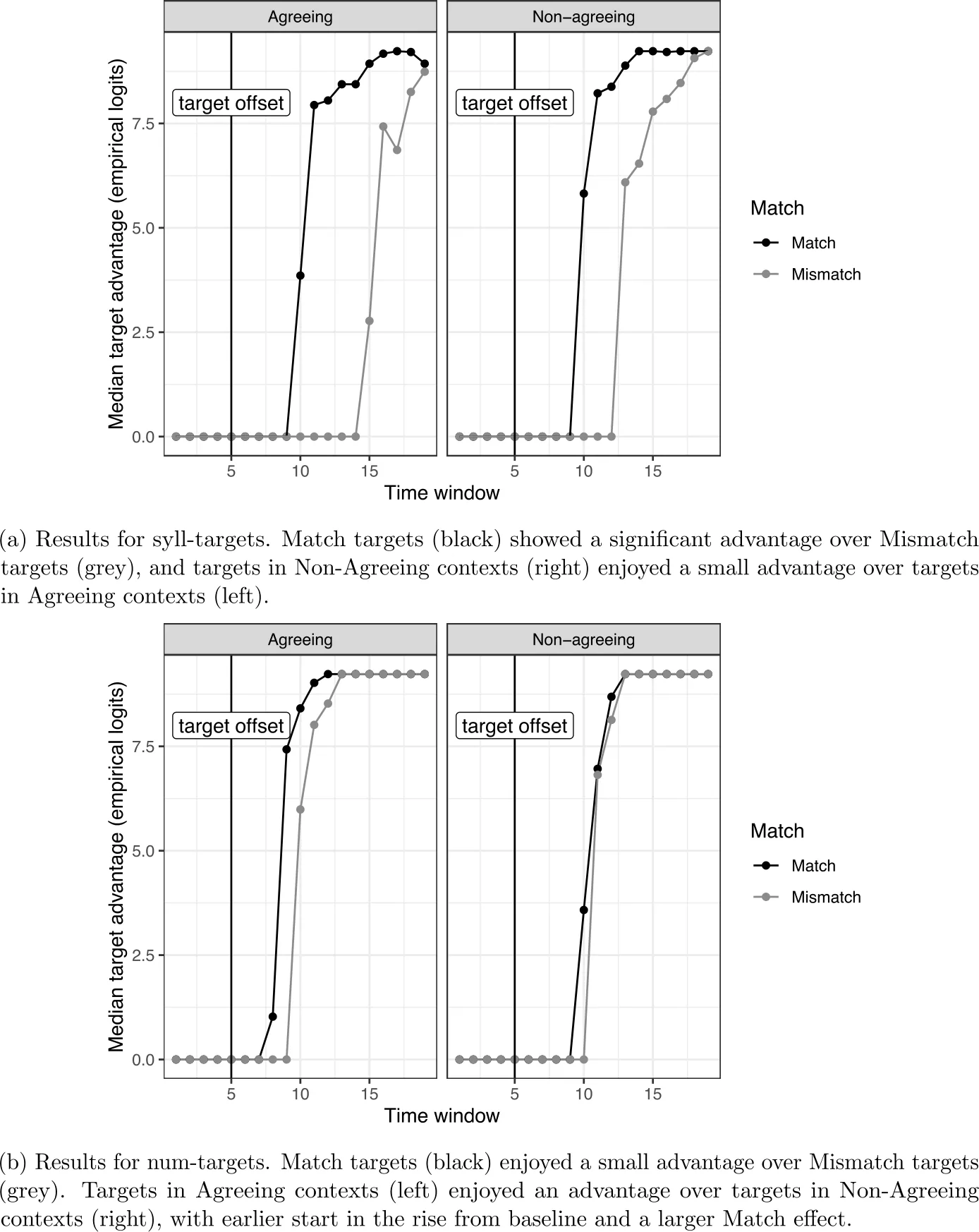
Median observed target advantage gaze traces Experiment 1, with English. Time window 5 anchors the offset of the target word in the audio stimulus

The model reflects a main effect of Match and Context. Target advantage was higher for Match targets than for Mismatch targets. This appears both in the parametric terms (*β* = 1.19, *p*<.001), and also in the smooth terms. As Fig. 4a shows, not only do the Match targets (black lines) enjoy an overall higher target advantage than Mismatch targets (grey lines), the shape of the curve is different. Match targets triggered an earlier, steeper increase in target advantage, followed by a levelling out in the last few windows. Mismatch targets show a later initiation of the rise in target advantage, and the later arrival at peak target advantage means they do not show the period of levelling out observed in the Match curves.

Interestingly, Agreeing contexts suffered slightly, compared to Non-agreeing contexts. This was a general pattern, as reflected in the parametric term (*β* = −0.55, *p*<.001), and did not affect the curve shapes.

#### Num-targets

The final model for num-targets, summarized in Table 4, included parametric terms for Context and Match, plus their interaction, and smooth terms for Match and Context, but no interaction. The parametric term for Match reflects a small advantage for Match targets over Mismatch targets (*β* = 0.14, *p* = .037), and the parametric term for Context reflects an advantage for Agreeing contexts over Non-agreeing ones (*β* = 0.56, *p*<.001). The positive parametric interaction between the two reveals that the effect of Match is stronger in Agreeing contexts than Non-Agreeing contexts. This effect can be observed in Fig. 4b, which shows the difference between black Match and grey Mismatch curves to be larger in the lefthand Agreeing context panel than the righthand Non-Agreeing panel. Indeed, so minimal is the difference between the Match and Mismatch curves in Non-Agreeing contexts, that it is likely the main effect of Match is driven solely by the interaction.

**Table 4:** Model summary of target advantage for looks to singular num-targets. Parametric intercept represents the overall median. The ‘baseline’ smooth represents the curve for Match, and Agreeing Context while the following terms give the difference smooths for the Mismatch curve and the Non-agreeing curve

*Parametric terms*				
Term	Estimate	Std. Error	*z*	*p*
(Intercept)	3.66	0.23	16.07	<.001
Match	0.14	0.07	2.08	.037
Context	0.56	0.07	8.44	<.001
Match × Context	0.34	0.13	2.64	.008
*Smooth terms*				
Term	Effective df	Ref.df	$\chi ^{2}$	*p*
Baseline	6.17	6.84	274.39	<.001
Match=Mismatch	4.76	5.77	42.38	<.001
Context=Non-Agreeing	4.88	5.89	48.83	<.001
(by-subject smooths)	166.52	299.00	1383.91	<.001
(by-item smooths)	421.43	909.00	2354.62	<.001

The smooth terms reveal additional differences in curve shapes. Match curves rise earlier and level off earlier than Mismatch curves; and the same is true for Agreeing Context curves compared to Non-Agreeing context curves. Note, in particular, that the Match curve in Agreeing contexts is the sole gaze trace that rises above 0 median target advantage less than four windows, or 200 ms, after target offset. We shall return to this point in the Discussion.

### Discussion

The results of Experiment 1 offer a straightforward *yes* in response to our first RQ. They confirm, first, that listeners use polysyllabic shortening to guide real-time perception. Participants better distinguished singular targets from polysyllabic competitors when those targets were lengthened than when they were inappropriately shortened. These findings are unsurprising, as they straightforwardly replicate the findings of multiple previous studies of this sort in both English (Cohen, 2024; Blazej & Cohen-Goldberg, 2015; Davis et al., 2002; Kemps et al., 2005b) and Dutch (Salverda et al., 2003; Shatzman & McQueen, 2006; Kemps et al., 2005a).

The findings also replicate Cohen (2024)’s observation that listeners use segmental compression to help them distinguish singulars from plurals, especially in Agreeing contexts, where the preceding determiner signals what sort of ancillary phonetic cue to expect. As Fig. 4 shows, in both syll-targets and num-targets, the median target advantage for the Match condition (black lines) consistently begins to rise above 0 by the fourth time window, or 200 ms, after the target offset. Since it takes about 200 ms to programme a saccade (Matin et al., 1993; Tanenhaus & Trueswell, 2006), this indicates that listeners begin to distinguish the target image from the competitor image in the Match condition just about as quickly as is physically possible. The only exception to this pattern is the curve for Match num-targets in Agreeing contexts, where the departure from 0 median target advantage begins earlier, at the second time window. This means participants were beginning to programme saccades to the target image even *before* the offset of the target word. In other words, listeners heard morphosyntactic agreement cues in the form of a singular determiner (*this/that*), which alerted them to the expected morphological form of the target word (unsuffixed singular). This expectation enabled listeners to benefit from the lengthened duration of the unsuffixed noun stem when it was present, to the point that they could turn their gaze to the correct, singular image even before they’d heard the unsuffixed ending of the word. By contrast, in the Non-Agreeing contexts, both Match and Mismatch curves do not depart from 0 target advantage until the fourth time window, or the minimum 200 ms programming time, after target offset confirmed the unsuffixed singular stem.

These findings speak to the generalizability of English listeners’ use of segmental compression. Here, we used General American English (GAE), while Cohen (2024) used Scottish Standard English (SSE). In principle, one might expect different results across the two dialects, as one key difference resides in SSE’s propensity to lengthen vowels before morpheme boundaries. This tendency is part of a larger phenomenon known as the Scottish Vowel Length Rule (SVLR, Scobbie et al., 2014). The SVLR is restricted in its application to high vowels and [ai] before voiced morphemes, but that does nevertheless describe multiple items used in this study, including *knee, bee, eye, tie*, and *fly*, all of which would be followed by a voiced allomorph of the plural *-s* suffix. This dialectal difference might be expected to weaken any effects of segmental compression in SSE listeners’ experience, relative to GAE listeners. Nevertheless both groups of listeners showed quite comparable effects: Following a shortened stem, both groups of listeners took longer to distinguish a singular target from its plural competitor than they did after lengthened stems.

Finally, the results also address the secondary research goal of determining whether the different sizes of Match effect reported by Cohen (2024) were an artefact of her stimulus construction. In that study, the num-targets and syll-targets bore an identical 80 ms Match manipulation, even though polysyllabic shortening tends to shorten syllables more than segmental compression. That identical manipulation may have produced a weaker Match effect for num-targets not because segmental compression is a weaker cue, but because its realization was too exaggerated. The results of this study, however, replicate Cohen (2024)’s findings. Here, the Match manipulation was designed to correspond to the expected acoustic differences between polysyllabic shortening and segmental compression, but that did not seem to offer any additional benefit to listeners in this experiment. The current results still show a weaker Match effect for num-targets than for syll-targets.

One possibility for this difference in Match effect size might relate to the structure of the following sentence. With syll-targets, the following word had onsets that were similar to the second syllable of the carrier competitor (e.g., *cart in front* resembles *carton* until the /f/). This stimulus design maximized the ambiguous period during which participants would need to rely on durational cues alone to distinguish target and competitor. With num-targets, however, it was not feasible to ensure that every word following the target began with /s/ or /z/. This meant that the ambiguous period was restricted to the stem alone, which could have limited the possible size of the Match effect. What is more, the following words’ onsets may have added some coarticulatory cues to the singular target stem. Since these cues would in most cases have been incompatible with a following /s/ or /z/, they may have undermined the misleading segmental compression in mismatch conditions that would otherwise have led the listener to expect a plural suffix. Thus, although it is possible that segmental compression is a less informative cue about word structure, compared to polysyllabic shortening, it is also possible that the design of the stimuli was not suited to give segmental compression the best chance to shine. A follow-up study that controls for the onsets of the words following the num-targets would be a useful way to distinguish between these possibilities.

In summary, Experiment 1 confirms that English listeners draw on multiple different durational cues to aid perception. When those cues are direct phonetic cues, such as polysyllabic shortening, listeners’ use of them does not seem to vary across morphosyntactic contexts. By contrast, with ancillary morphophonetic cues such as suffix-triggered segmental compression, listeners use the cues more when they are supported by surrounding morphosyntax. With this baseline for comparison established, we can now examine how Spanish listeners, who speak a language with pervasive morphosyntactic agreement, use segmental compression as a cue to word structure across different syntactic contexts.

## Experiment 2

Experiment 2 transferred Experiment 1 into Spanish as faithfully as possible, in order to answer RQs 2-4. It is therefore worth reviewing those RQs and predictions. First, if Spanish listeners use segmental compression (RQ2), they should show a Match effect for num-targets. By comparing any Match effect for num-targets in Exp 2 with the observed Match effect for num-targets in Exp 1, we can determine whether Spanish listeners show larger or smaller effects of segmental compression than English listeners (RQ3). Finally, if Spanish-English bilinguals are more sensitive to these match effects than listeners with less bilingual language experience, then we should find that greater English proficiency predicts stronger Match effects (RQ4).

### Methods

#### Participants

Participants were recruited from the student population and surrounding community of the Universitat Rovira i Virgili in Tarragona, Spain, and compensated €15 for participation. This region of Spain was selected because it does not participate in the aspiration of coda /s/ which characterizes so many other varieties of Spanish (Núñez-Méndez, 2022), and so its treatment of final plural *-s* is a good match to the situation in English. In total, we recruited 61 participants, selected from two groups based on the results of a screening questionnaire. High-proficiency Spanish-English bilinguals (*n* = 26) were recruited from those who self-reported English proficiency in reading, writing, speaking, and listening at a score of 5 or better on a 7 point scale, or had completed or were currently studying at the C1 level of English. Since it was tricky to find listeners who lacked any proficiency in English in the same general recruitment population, we instead decided to recruit a second group of low-proficiency bilinguals (*n* = 35). This category contained participants whose self-reported proficiency scored 3 or below on the same scale, and had not advanced beyond level B2. Details of their self-ratings are given in Table 5, along with details of some standardized measures of proficiency, which confirmed the desired distinction between the two groups. As the bottom two blocks of the table show, both groups had very similar scores in measures of Spanish fluency, while the high-proficiency bilinguals scored roughly twice as high as the low-proficiency bilinguals in all three measures of English proficiency.

**Table 5:** Participant details for high-proficiency Spanish-English bilinguals and low-proficiency Spanish-English bilinguals. The second block contains self-reported ratings on a scale of 1-7 for Spanish; the third block contains ratings for English. The lower two blocks give the standardized test scores for verbal fluency, Mint naming test, and DELE or MELICET grammar tests, Spanish first and English in the lowest block

Statistic	High-prof (n = 26)		Low-prof (n = 35)	
	Mean	(SD)	Mean	(SD)
age (years)	21.76	(3.82)	22.47	(2.73)
lg. learning ability	5.54	(0.59)	3.38	(1.36)
speaking (Span)	6.88	(0.33)	6.88	(0.42)
listening (Span)	7.00	(0.00)	6.94	(0.25)
reading (Span)	6.96	(0.20)	6.95	(0.21)
writing (Span)	7.00	(0.00)	6.75	(0.57)
speaking (Eng)	5.38	(0.50)	2.43	(1.00)
listening (Eng)	5.77	(0.65)	3.13	(1.12)
reading (Eng)	5.77	(0.71)	3.67	(1.08)
writing (Eng)	5.38	(0.80)	2.92	(1.11)
verbal fluency (Span)	12.48	(4.60)	11.82	(4.22)
Mint score (Span)	94.39	(3.13)	91.61	(3.64)
DELE score (%)	89.08	(5.22)	84.00	(9.51)
verbal fluency (Eng)	9.38	(4.18)	5.62	(3.09)
Mint score (Eng)	62.39	(14.85)	32.02	(9.17)
MELICET score (%)	68.77	(15.17)	27.71	(9.76)

Because the experiment took place in the Catalonia region of Spain, almost all participants were highly proficient in Catalan as well as Spanish. Catalan proficiency was not controlled for, because the key properties of Spanish relevant to this study are shared with Catalan. Like Spanish, Catalan enjoys near-universal determiner-noun number agreement. Further, observed bilingual advantages over monolinguals in phonetic and phonological processing also seem to separate multilinguals from bilinguals (Tremblay & Sabourin, 2012).

#### Materials

Similarly to Experiment 1, critical stimuli were built around a set of 71 stems appearing in three forms: a singular target word (e.g., *cama*, ‘bed’), a plural (e.g., *camas*, ‘beds’), and a carrier (e.g., *cámara*, ‘room’). The inclusion of carrier words ensures that Experiment 2 is structurally as similar to Experiment 1 as possible. We do not analyse the results of syll-targets here because the stress patterns between the singulars and carriers were not constant, and so manipulating duration would produce a confound in the design. A shortened stem might indicate the presence of polysyllabic shortening, or it might indicate that stress had shifted off of it. Both of these cues serve to signal that further syllables follow, and are thus confounded.

The selection of our stimulus triplets ensured maximum comparability to Experiment 1, but brought with it constraints on the number of suitable singular targets. Each stem needed to be imageable, so as to be suitable for a visual-world paradigm. Stems were further phonologically constrained to be a two-syllable noun ending in a vowel, so that the addition of the plural suffix would not add an additional syllable. These constraints resulted in 71 singular/plural/carrier triplets, which is less than the 91 stems used in Experiment 1. This has the effect of reducing the statistical power of our results — although this reduction is offset by the larger number of participants we recruited.

Each stem could appear in one of two sentence types, as in Experiment 1: an Agreeing context, in which the target word was preceded by an agreeing determiner (5), and Non-Agreeing context, in which the target word appeared as a metalinguistic reference, and thus was not preceded by anything which could signal its number (6).

(5)**Figure:**
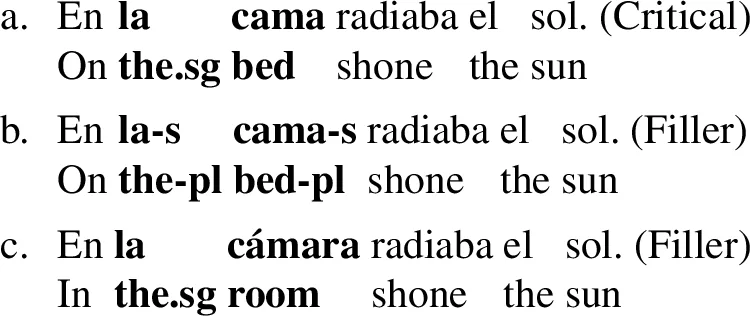
(6)**Figure:**
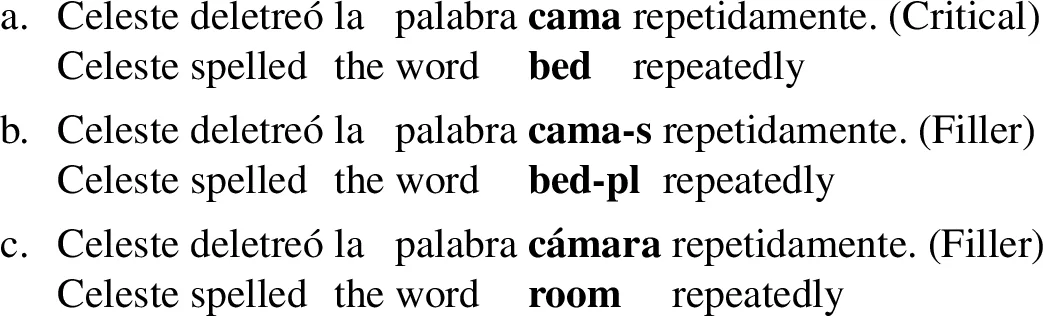

As in Experiment 1, the phonetic context following singular targets was designed to mimic the phonetic material characterizing the carrier competitors. Thus, the target *cama*, ‘bed’, was followed by *radiaba*, ‘shone’, in the Agreeing contexts in (5a), because *radiaba* begins with [ra], which is also the post-stem syllable in the carrier competitor, *caḿara*, ‘room’. It was not always possible to place a fully ambiguous following syllable in this way, given the constraints on sentence plausibility. Thus, in (6a), the following word *repetidamente*, ‘repeatedly’, begins with [re], not [ra]. Still, even in this case the following [r] serves to introduce a little temporary ambiguity into the post-target portion of the sentence.

In the case where the carrier word was of a different gender from the singular word, the agreeing determiner was a possessive (e.g., *mi(s)*, ‘my’, *su(s)*, ‘his/her’), which agrees in number but not gender. If the gender of the singular word and carrier were the same, then there were no restrictions on which determiner carried the number information. These precautions ensured that the determiner always signaled the number of the following noun, distinguishing singulars and plurals, but never signaled whether the target would be singular or carrier. The full list of stimuli is included in our OSF archive for this paper: https://osf.io/u7y6z/overview?view_only=716487e43450451ab3d6db240ddf17f4.

All recordings were produced by a phonetically trained male native speaker of Castilian Spanish, who took pains to ensure similar intonation across the preambles of corresponding sentences, to facilitate splicing. Before manipulating the durations of the noun stems, we examined their raw durations to see if there was evidence of any underlying segmental compression. As shown in Table 6, there was a small numerical difference in stem durations between singulars and plurals, in the direction consistent with segmental compression: stems in suffixed plurals were shorter than stems in unsuffixed singulars. As with the English recordings, we cannot be sure whether the small size of these differences reflects naturalistic speech patterns in this variety of Spanish, or instead results from a conscious attempt to minimize prosodic differences across sentences containing the same items.

**Table 6:** Mean stem durations (and standard deviations) in ms from the raw recordings of Spanish stimuli, along with the durational difference from the singular counterpart

Context	singular	plural	
Agreeing	290.1 (40.0)	282.9 (39.0)	
dur. dif. from sg.	0	−7.7	
Non-agreeing	343.4 (47.6)	340.9 (39.6)	
dur. dif. from sg.	0	−2.5	

Stem durations were manipulated in the identical manner as in Experiment 1, where *stem* here means both syllables of the noun. Although the magnitude of any apparent segmental compression in our raw recordings was much smaller in Spanish (Table 6) than in English (Table 1), we elected to keep the manipulations equivalent to ensure that our results allowed us to make meaningful comparisons across the two Experiments. Otherwise, any differences between English and Spanish listeners would be confounded with the differences in the durational manipulation across their respective experiments.

#### Design

All sentences, experimental lists, and configurations of target, competitor, and distractor pictures were constructed in the same manner as Experiment 1.

#### Procedure

Paralleling the procedure in Experiment 1, participants began by giving informed consent, and then proceeded directly to the eye-tracking experiment. As much as possible all interactions were in Spanish, although some of the high-proficiency bilinguals, upon hearing the experimenter’s accent and grammar, spontaneously switched to English.

The procedure of the eye-tracking experiment was identical to Exp 1, except that the different number of critical stimuli led to a different block size. After three practice trials, participants completed the full set of 142 sentences, split into four blocks of 35, 35, 35, and 37.

After the eye-tracking portion was completed, participants completed a language background questionnaire, and then a set of proficiency tests, first in Spanish, and then in English. The first proficiency test was the MINT naming task (Gollan et al., 2012), followed by a verbal fluency task, followed by a grammar test adapted from the test used by Diplomas of Spanish as a Foreign Language (DELE: *Diplomas de Español como Lengua Extranjera*). These tasks were then repeated in English. The naming task and verbal fluency used the same materials in Spanish and English, while the English version of the grammar task was adapted from the Michigan English Language Institute College Entrance Test (MELICET). From start to finish, the entire procedure took about 2 hours.

#### Analysis

The resulting gaze traces for num-targets were analysed in the same way as Experiment 1. Gaze traces were binned, converted to empirical logits, and target advantage for each bin was calculated by subtracting looks to the competitor from looks to target. Median target advantage was then analysed using QGAMs. Variables included Match (Match vs. Mismatch) and Context (Agreeing vs. Non-agreeing), as in Experiment 1, with the added variable of English Proficiency (High vs. Low), as well as their interactions. In the parametric terms, these factors were all effects-coded, with Match, Agreeing Context, and High English Proficiency set as 0.5, and Mismatch, Non-Agreeing Context, and Low English Proficiency set as −0.5. The smooth terms were treatment-coded as ordered factors, with the default level as Match, Agreeing Context, and High English Proficiency. Both the num-target and syll-target models were built to include all interactions from the start, as the research questions defined by this study ask whether English proficiency modulates listeners’ use of segmental compression across different syntactic contexts. They were then simplified through backward elimination of terms as in Experiment 1.

### Results

#### Num-targets in Spanish

**Fig. 5:**
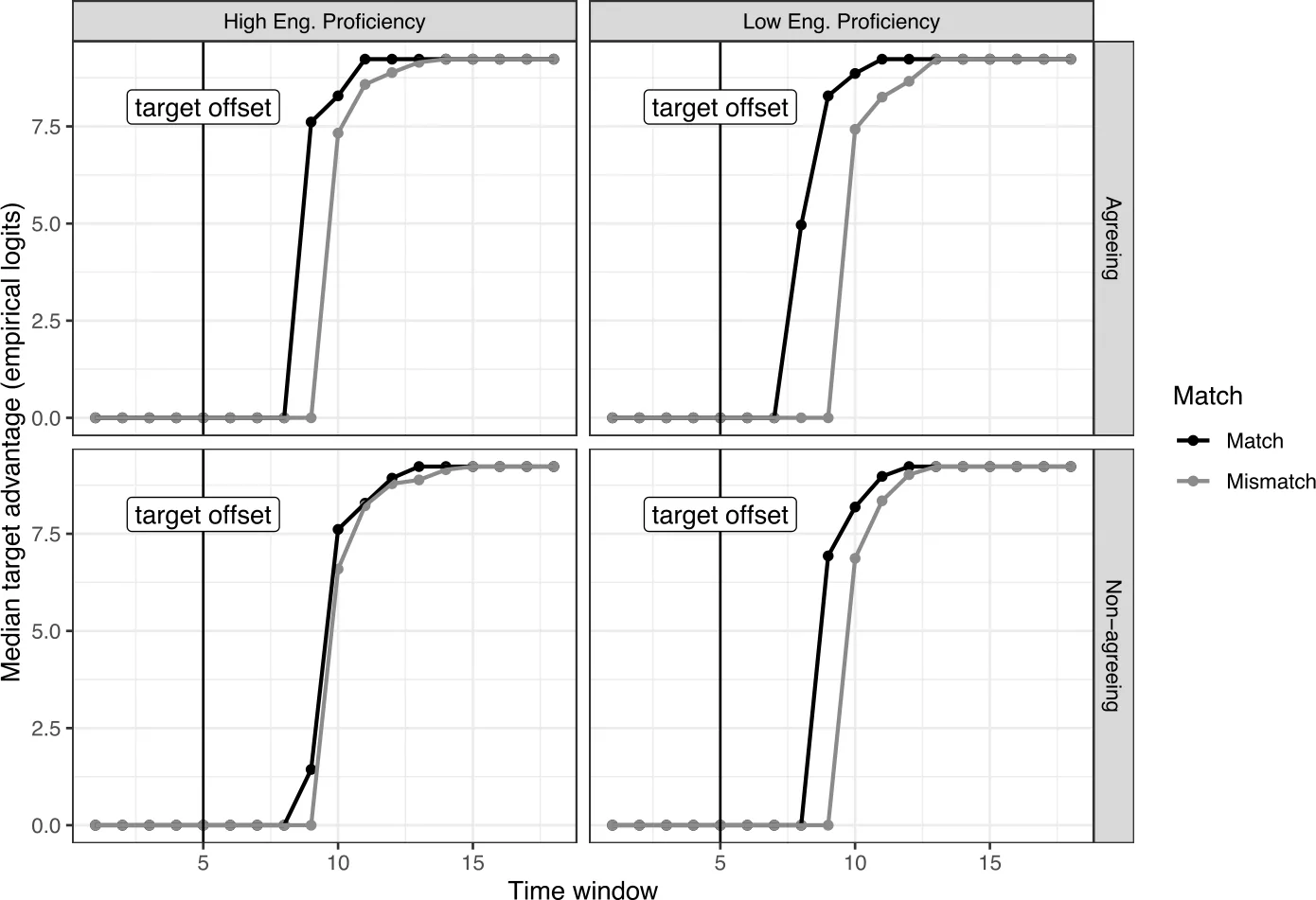
Results for num-targets in Spanish (Experiment 2). Match targets (black) enjoyed a small advantage over Mismatch targets (grey), which was larger in Agreeing contexts than Non-Agreeing contexts. These patterns were not significantly different across values of English Proficiency

The final model, summarized in Table 7, contained parametric terms for Match, Context, and their interaction, and smooth terms for Match and Context alone. No effect of English Proficiency emerged. The main effect of Match reflects a small advantage for Match over Mismatch (*β* = 0.44, *p*<.001), and the main effect of Context reflects an advantage of Agreeing over Non-Agreeing Contexts (*β* = 0.33, *p*<.001). The positive interaction term indicates an enhanced effect of Match in Agreeing Contexts compared to Non-agreeing Contexts. The smooth terms reflect different curve shapes for Match and Mismatch conditions. As Fig. 5 illustrates, Match targets (black lines) elicited earlier rises in target advantage, and earlier arrival at peak target advantage, than Mismatch targets (grey lines). The same is true for targets in Agreeing contexts (top panels) compared to those in Non-Agreeing contexts (bottom panels). The apparent differences between High English Proficiency (left) and Low English Proficiency (right) did not reach significance during modeling.

**Table 7:** Model summary of target advantage for looks to singular num-targets in Experiment 2, Spanish. Parametric intercept represents the overall median, while parametric terms represent main effects and interactions. The ‘baseline’ smooth represents the curve for Match in Agreeing Context. The following terms gives the difference smooth for Mismatch targets, and Non-agreeing Context. No effect of English Proficiency emerged

*Parametric terms*				
Term	Estimate	Std. Error	*z*	*p*
(Intercept)	3.80	0.19	20.43	<.001
Match	0.44	0.06	7.57	<.001
Context	0.33	0.06	5.74	<.001
Match × Context	0.37	0.11	3.23	.001
*Smooth terms*				
Term	Effective df	Ref.df	$\chi ^{2}$	*p*
Baseline (Match, Agreeing)	7.12	7.83	610.14	<.001
Mismatch	5.17	6.22	61.92	<.001
Non-agreeing	2.25	2.79	19.51	<.001
(by-subject smooths)	283.58	609.00	1760.63	<.001
(by-item smooths)	285.57	709.00	1767.35	<.001

#### Num-targets compared across English and Spanish

**Fig. 6:**
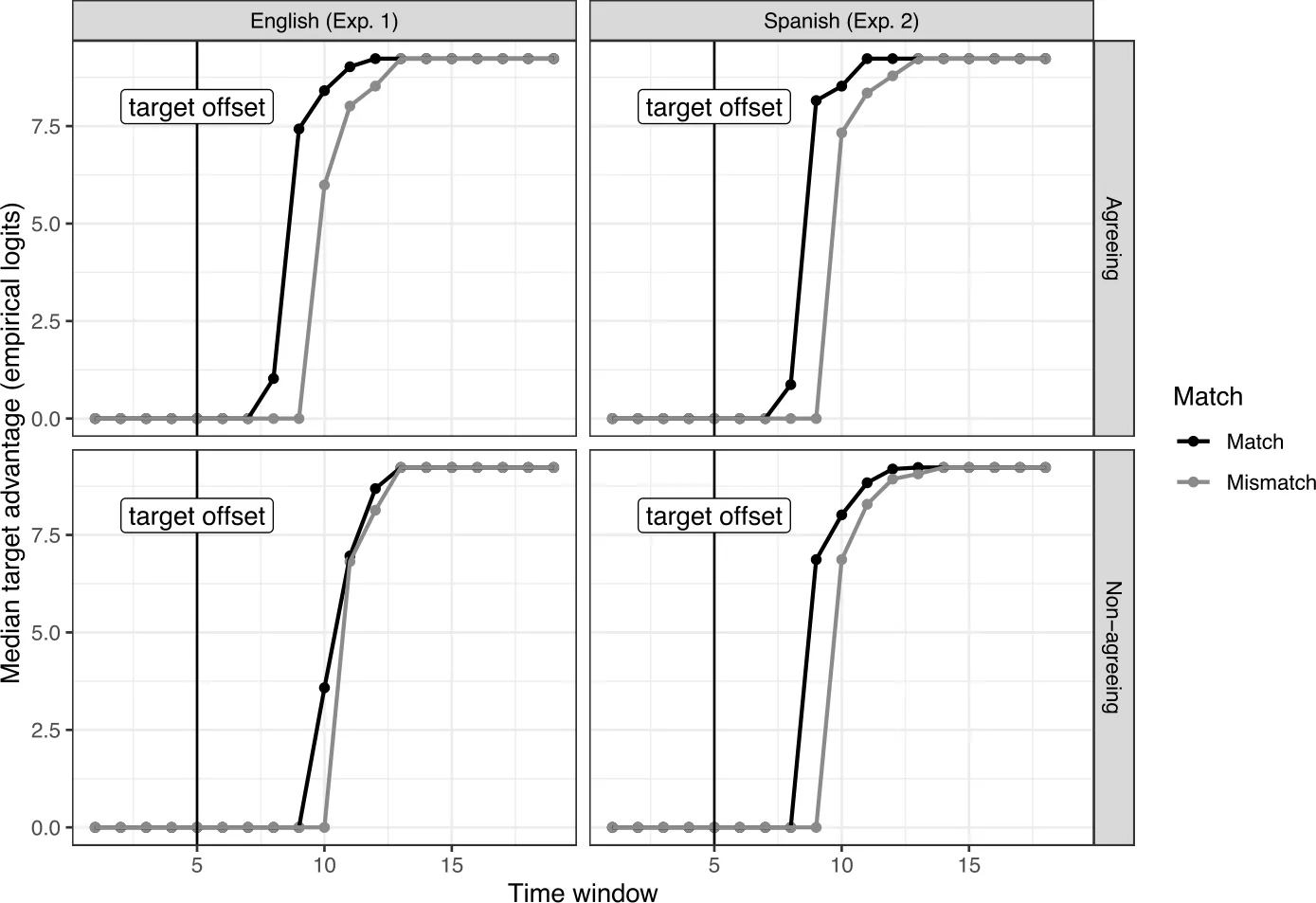
Comparison of results across English (left) and Spanish (right) num-targets. Match targets (black) showed a significant advantage over Mismatch targets (grey), with a slightly larger separation for Spanish stimuli than English stimuli

As a final analysis, we combined the English and Spanish results into a single dataset and built a model directly comparing the size of the Match effect in the two languages. The final model for num-targets in both English and Spanish ended up with the equivalent structure — parametric terms for Match, Context, and their interaction, along with smooth terms for Match and Context alone. Therefore, we built the combined model upon that structure, to which we added an interaction between Match and Language in both the parametric and smooth terms. Match and Context remained effects-coded in the parametric terms, as before. Language, however, was treatment-coded in the parametric terms, with English as the baseline. This ensured that the coefficients represented a direct comparison of the effect of Match in English and Spanish. All variables were treatment-coded as ordered factors in the smooth terms. The summary for this model is shown in Table 8, with the relevant interactions visualised in Fig. 6.

**Table 8:** Model summary of combined analysis of target advantage for looks to singular num-targets across Experiment 1 (English) and Experiment 2 (Spanish). Parametric intercept represents the overall median, while parametric terms represent main effects and interactions. The ‘baseline’ smooth represents the curve for English Match in Agreeing Context. The following terms give the difference smooths for the other combinations of Language and Match, and Non-agreeing Context. No effect of English Proficiency emerged

*Parametric terms*				
Term	Estimate	Std. Error	*z*	*p*
(Intercept)	3.54	0.22	16.06	<.001
Match	0.14	0.07	2.09	<.05
Context	0.44	0.04	9.90	<.001
Match × Context	0.36	0.09	4.25	<.001
Language = Spanish	0.31	0.29	1.04	.30
Match × Language = Spanish	0.29	0.09	3.24	<.01
*Smooth terms*				
Term	Effective df	Ref.df	$\chi ^{2}$	*p*
Baseline (Match, Agreeing, English)	7.19	7.80	385.99	<.001
Mismatch, English	4.67	5.64	39.44	<.001
Match, Spanish	4.19	4.97	10.70	<.05
Mismatch, Spanish	3.30	3.87	6.01	.24
Non-agreeing	5.41	6.59	45.85	<.001
(by-subject smooths)	446.68	908.00	3109.93	<.001
(by-item smooths)	688.43	1618.00	3903.25	<.001

As the parametric terms show, Match enjoyed a significant interaction with Language. For English, the size of the Match effect is only 0.14 empirical logits of target advantage (*p*<.05). This corresponds to the Match effect that we already observed in the Results for Experiment 1 (Table 4). For Spanish, however, Match has a significantly larger effect, exceeding the effect in English by 0.29 empirical logits (*p*<.01). This yields a Match effect size of 0.44 (with rounding) for Spanish, which corresponds to the effect observed in the Spanish-only analysis in Table 7.

The smooth terms reveal slightly different curve shapes across the different values of Match and Language. The curve for Spanish Mismatch trials does not differ from the baseline curve of English Match trials (*p* = .24), while English Mismatch does differ from that baseline (*p*<.001), as does Spanish Match (*p*<.05). The details of those curve shapes, however, are of less interest than the parametric interaction, which confirms the significantly larger overall size of the Match effect in Spanish than in English.

### Discussion

Spanish listeners in Experiment 2 produced a larger Match effect when nouns were preceded by an agreeing determiner, compared to when they were presented in a context of metalinguistic mention, without any determiner. This is the same pattern observed in Cohen (2024), and replicated in Experiment 1 above. The mechanism behind this interaction in English, as Cohen (2024) proposes, comes from listeners’ ability to use the preceding agreeing determiner to predict not only the morphological form of the following noun, but also the phonetic realisation of that morphological form. This phonetic prediction allows them to react more quickly when the prediction is confirmed in the actual word’s production. If this same mechanism is at work in Spanish listeners, then that means not only that they use segmental compression to distinguish singular from plural nouns; but also that they use that segmental compression in a morphosyntactically informed way. In other words, segmental compression operates as an ancillary morphophonetic cue in Spanish, reliably enough that listeners use it in real time speech perception.

It is worth briefly considering an alternative explanation for this interaction. It relates to the naturalness of the stimuli. The Non-Agreeing context was built through slightly awkward constructions, usually organized around someone spelling, learning, or pronouncing a word: *Celeste deletreó la palabra* cama *repetidamente*, ‘Celeste spelled the word *bed* repeatedly’. If participants were taken aback by these slightly awkward sentence structures, they might have had less working memory to devote to interpreting the durational manipulation, resulting in a weaker Match effect in the less natural Non-agreeing context.

However, there are two counterarguments to this account. First, if the awkwardly constructed Non-agreeing sentences truly interfered with listeners’ ability to interpret the durational manipulation, then we would have expected a similar interaction with syll-targets – i.e., those singular targets whose competitors were multisyllabic carrier versions. We did not present the results of that analysis here, as it relates to listeners’ ability to detect cues to stress location, rather than direct phonetic cues to polysyllabic shortening, and so is not relevant to our research questions. Nevertheless, we did conduct such an analysis, and found no interaction between the Match effect and sentence context. With syll-targets, the effect of Match was consistent across both contexts.

The second counterargument lies in the similarity between the interaction observed here with num-targets and the equivalent interaction observed in Experiment 1, as well as Cohen (2024). The awkward metalinguistic mention structure applies equally to Experiment 1 stimuli as it does to Experiment 2 – but crucially it did not appear in Cohen (2024)’s stimuli. Those stimuli manipulated context with a straightforward swap between agreeing *this/that* and non-agreeing *the*. Yet Experiment 1 here closely replicated Cohen (2024)’s findings. This suggests that, in English at least, the awkwardness of the metalinguistic mention structure was not responsible for generating the interaction, since the same interaction was present in experiments with and without the awkwardness. It would be an uphill battle then, to argue that the metalinguistic mention structure was not responsible for that interaction in the two English experiments, but nevertheless was responsible for producing that identical interaction in Spanish – especially when it is so much simpler to argue that the same effect is present in all three experiments because the same mechanism is at work.

We are comfortable, therefore, in concluding that Experiment 2 straightforwardly replicates Experiment 1’s findings because both Spanish and English employ the same perceptual strategies. Spanish and English listeners both use morphosyntactic agreement to form predictions about which ancillary morphophonetic cues to expect, and guide their perceptual strategies accordingly.

## General discussion

In two experiments, we tested whether English and Spanish listeners attend to different types of cues that signal word structure in real-time speech perception. Direct phonetic cues reflect purely phonological features. Ancillary morphophonetic cues are a subset of direct phonetic cues, because the phonological processes that triggered them were the result of morphological processes. Here, we examined polysyllabic shortening as a direct phonetic cue to a word’s syllable count in English; and segmental compression as an ancillary morphophonetic cue to a noun’s grammatical number in both English and Spanish. We compare English and Spanish because they both allow suffixation in *-s* as a plural marker without changing the syllable count of the noun, but unlike English, Spanish consistently signals noun number with morphosyntactic agreement elsewhere in every noun phrase. Its grammatical structure therefore robustly reinforces segmental compression as a cue to the noun’s grammatical number, which may in turn affect how strongly listeners attend to that ancillary morphophonetic cue in speech perception.

The results reported above allow us now to answer our research questions.

### Four answers to four questions

**RQ1:** Do listeners draw on direct phonetic cues and ancillary morphophonetic cues when perceiving English noun phrases, and does morphosyntactic context affect that process? In other words, can we replicate Cohen (2024)’s findings?

Yes: in Experiment 1, English-speaking listeners showed a large Match effect for syll-targets, indicating robust use of polysyllabic shortening as a direct phonetic cue. This is also consistent with other previous work in English (Blazej & Cohen-Goldberg, 2015; Davis et al., 2002; Kemps et al., 2005b; Cohen, 2024) and Dutch (Kemps et al., 2005a; Salverda et al., 2003; Shatzman & McQueen, 2006).

Listeners also showed a smaller Match effect for num-targets, indicating use of segmental compression as an ancillary morphophonetic cue. This effect was smaller than the effect of polysyllabic shortening. Conceivably it might reflect the fact that the durational manipulation for num-targets was smaller than the durational manipulation for syll-targets. However, since Cohen (2024) found the same difference in effect sizes while using an identical durational manipulation of 80 ms for both syll-targets and num-targets, this is unlikely. Alternatively, it may reflect the fact that we did not control the phonological context following num-targets the way we did following syll-targets, which constrained the possible size of the segmental compression effect.

The possibility that the weak effect of segmental compression is merely an artefact of stimulus design is plausible and entirely testable in future work. But it is also a bit dull. We shall therefore indulge in some speculation regarding more interesting reasons for the weaker effect of segmental compression. First, segmental compression may simply be a weaker cue to word structure. It distinguishes words that do not vary in syllable count, and applies only to a subpart of a single syllable. Targets of segmental compression may therefore have less room to expand and contract in duration. Further, the segmental compression caused by suffixation might be weakened by the pre-boundary lengthening that can accompany morphological boundaries (Sugahara & Turk, 2009).

Second, as proposed by Cohen (2024), segmental compression as a cue to plurality does less work in disambiguating potential meanings than polysyllabic shortening. The difference between *cart* and *carton*, or between *cama*, ‘bed’, and *cámara*, ‘room’, is a matter of entirely different lexical items. The difference between *cart* and *carts*, or *cama*, ‘bed’, and *camas*, ‘beds’, is a matter of grammatical number alone. Less confusion will result from temporarily confusing a singular for a plural, than confusing two entirely different words. Accordingly, listeners may simply not consider segmental compression as valuable a perceptual cue as polysyllabic shortening.

Finally, listeners showed an interaction between Match and morphosyntactic context: After an agreeing determiner, listeners were more sensitive to segmental compression than in contexts without any number agreement information. These results also match those reported by Cohen (2024), confirming that our experimental design is sound, and that the effect of interest – the interaction between Match and Context – is replicable.

**RQ2:** Do Spanish-speaking listeners use segmental compression in noun stems to perceptually distinguish unsuffixed singular nouns from suffixed plural nouns?

Yes: Spanish listeners were sensitive to segmental compression. They more quickly distinguished the target singular noun from its plural competitor when the noun stem was lengthened – consistent with an unsuffixed singular noun – than when it was shortened.

Production studies have offered conflicting data about whether the presence of a coda shortens syllable nuclei in Spanish. Studies on Altiplateau Mexican Spanish and Southern Chilean Spanish report that it does (Marchini & Ramsammy, 2022a,b). Other studies find no such effect (Marín Gálvez, 1994; Aldrich & Simonet, 2019). However, these null effects may arise from other sources. Marín Gálvez (1994) used only two speakers, so perhaps their results reflect a lack of statistical power to detect segmental compression, rather than an absence of segmental compression itself. Aldrich and Simonet (2019) used more speakers, but did not control for the dialects, and indeed report substantial dialectal variability in their speakers, with both Mexican and European Spanish, among others, represented. Since Marchini and Ramsammy (2022a,b) report that some syllable-structure effects on vowel duration differ across dialects, the dialectal variation in Aldrich and Simonet (2019)’s speaker groups might have obscured any segmental compression effects. A corpus study would offer clearer evidence about the presence of segmental compression, but as no such study exists (yet), we must draw inferences from imperfect data about whether segmental compression is a speech feature that Spanish listeners know to draw upon during speech perception. The results from this study suggest that it may be — at least for the variety of Spanish studied here.

A limitation on this conclusion relates to the size of the Match effect in Experiment 2. As suggested in Table 6, the magnitude of a segmental compression effect that listeners use as a speech cue in Spanish may well be quite small. The raw recordings from the speaker in this study produced a segmental compression effect on the order of 7 ms. However, Marchini and Ramsammy (2022b) report much larger effects in Altiplateau Mexican Spanish, on the order of 20-30 ms in vowel duration alone. Yet even 30 ms of compression is smaller than the magnitude of the Match manipulation we built into our experiment. It is possible that our results reflect not listeners’ natural use of segmental compression as a cue, but rather their ability to draw upon an exaggerated cue in laboratory conditions. Just as further work on production is necessary to confirm the magnitude of segmental compression across dialects of Spanish, further work subsequent to that will be necessary to explore listeners’ use of a cue that is more precisely aligned with its status in natural speech.

**RQ3:** Do Spanish listeners differ from English listeners in their perceptual sensitivity to segmental compression in noun stems across different morphosyntactic contexts?

Yes. Whereas English listeners showed a main effect of Match of only 0.14 empirical logits, Spanish listeners had an effect three times as large, at 0.44 empirical logits. We can propose three possible reasons why it might be so much greater.

The first is that our Spanish listeners in Experiment 2 were almost all at a minimum bilingual in Spanish and Catalan, with additional experience in English. By contrast, most of the English listeners in Experiment 1 were monolingual. Since bi/multilingualism shows benefits for phonetic processing (Zhao et al., 2023; Spinu et al., 2018; Antoniou et al., 2014; Tremblay & Sabourin, 2012), the advantage of our Spanish listeners in using segmental compression to identify num-targets may simply reflect their multilingual backgrounds.

The second reason relates to the different coda structures used in the two languages. In Experiment 1, single-syllable English nouns were pluralized with the addition of a single-segment suffix, *-s*. This could be added to open-syllable stems (e.g., *tie, bee, crow*), closed syllable stems with simple codas (e.g., *bag, sock, pen*), or closed syllable stems with complex codas (e.g., *lamp, monk, hand*). By contrast, the only way to pluralize Spanish nouns without changing their syllable count was to use nouns that ended in open syllables only (e.g., *coco*, ‘coconut’; *cama*, ‘bed’). Increasing the complexity of an existing coda in English does not shorten the stem duration as reliably as closing an open syllable (Katz, 2012; Marin & Pouplier, 2010). Possibly, then, the English num-targets simply lacked the phonological structure that allowed segmental compression to shine forth in its full potential, and listeners correspondingly exploited that cue less.

However, if that were the case, then we’d expect more segmental compression when adding the plural suffix to stems with open syllables than stems with simple or complex closed syllables. Experimental work in this domain has been contradictory, and the materials used do not directly bear on our particular case. Munhall et al. (1992) found segmental compression when /s/ was added to a syllable ending in voiceless plosives, and Shaiman (2001) replicated that finding with syllables ending in /p/. However, neither Katz (2012) nor Marin and Pouplier (2010) found any segmental compression when voiced /z/ was added to a syllable ending in a nasal; and lest the attentive reader hypothesize that the difference reflects voicing of the final coda, we hasten to add that Marin and Pouplier (2010) also found no compression when *-s* was added to syllables ending in /p/ or /k/. None of these studies, however, compared the effect of adding /s/ or /z/ to open syllables with the effect of adding those segments to closed syllables; and Cohen and Carlson (2024)’s corpus study did not include syllable structure in its analysis. It remains unclear, therefore, whether segmental compression is actually more extreme when open syllables become closed, as opposed to when simple codas become complex. Without that evidence from production, it is less convincing to argue that listeners are drawing on such patterns in perception.

The third, most interesting, possibility for the different Match effects in English and Spanish listeners springs from differences in morphosyntax across the two languages. Recall that the Match effect was stronger in Agreeing contexts compared to Non-Agreeing contexts for both languages. Cohen (2024) proposes that a preceding agreeing determiner helps listeners predict the number of the upcoming noun, and hence its likely phonetic realization, positioning them to benefit more when that prediction is confirmed. Spanish nouns are almost universally preceded by some kind of morphosyntax signalling their number, so Spanish listeners will have more opportunity to learn this ancillary morphophonetic cue, deploy it, and fine-tune their use of it. Perhaps, then, Spanish listeners are more skilled at this perceptual task than English listeners.

**RQ4:** Do proficient Spanish-English bilinguals show greater sensitivity to segmental compression than listeners with less English experience?

We have no evidence of this: Although Fig. 5 shows an intriguing pattern whereby listeners with less English proficiency seem slightly more sensitive to segmental compression than listeners with high English proficiency, this apparent difference did not emerge in the statistical analysis. We cannot, therefore, conclude that there is any effect of proficiency on listeners’ perceptual processing.

In the Introduction, we hypothesised two possible mechanisms that would have predicted such an interaction. First, we proposed Spanish listeners might import their experience interpreting English phonetic cues into Spanish, as they have been shown to do in other perceptual domains (Carlson et al., 2016; Carlson, 2018). Second, we proposed that greater multilingual experience might improve overall perceptual acuity (Antoniou et al., 2014; Spinu et al., 2018; Tremblay & Sabourin, 2012; Zhao et al., 2023). Both mechanisms – which are not mutually exclusive – would predict larger Match effects in listeners with greater English proficiency. The lack of any such interaction between Match and English proficiency fails to support either hypothesis.

Possibly that lack of significance reflects a simple lack of statistical power to detect an interaction involving an already small effect. However, absent evidence from a larger-scale, higher-powered study, we have no reason to believe that multilingual experience affects Spanish listeners’ ability to use segmental compression during real-time sentence processing.

### How to compare direct phonetic and ancillary morphophonetic cues

In Experiment 1, ancillary morphophonetic cues – i.e., segmental compression – produced a smaller match effect than the direct phonetic cues of polysyllabic shortening. Since the specific phonetic process that generates these two types of cues is not the same, it is difficult to draw any conclusions about whether there is any appreciable difference in the relative contribution of these two cue types to online speech perception. Yet such a direct comparison is possible, in principle.

One way to compare the relative strength of direct phonetic cues with ancillary morphophonetic cues would be to design a study similar to the experiments described here, with the key difference being that both plural and carrier competitors have two syllables. In English, nouns ending in sibilants, such as *badge*, acquire a second syllable in the plural (*badges*). By comparing how listeners use polysyllabic shortening to distinguish *badge* and *badger*, compared to *badge* and *badges*, we could distinguish the role of both cues in perception. In both cases, polysyllabic shortening would serve as a cue to distinguish *badge* from its competitor; but with *badge/badger*, the cue would be a direct phonetic cue, while with *badge/badges* it would be an ancillary morphophonetic cue.

A similar study could be constructed to examine segmental compression in this way. English offers some classic examples of morphemic and non-morphemic *-s* in triplets such as *tack, tacks*, and *tax*; or *lap, laps* and *lapse* (Walsh & Parker, 1983; Losiewicz, 1992). Segmental compression would serve as an ancillary morphophonetic cue to distinguish singular *lap* from plural *laps*, and as a direct phonetic cue to distinguish *lap* from *lapse*.

If listeners distinguish between direct phonetic cues and ancillary morphophonetic cues that arise from the same process, such as polysyllabic shortening or segmental compression, there are two differences that we can predict for such a study. First, we should find an interaction between Match and Context for *lap/laps* or *badge/badges*, replicating the findings here and in Cohen (2024). However, there should not be such an interaction for *lap/lapse* or *badge/badger*. With *lap/laps* or *badge/badges*, listeners should make more use of the durational cue after agreeing determiners than after non-agreeing determiners, because the agreeing determiners signal which morphological form follows. Therefore, the determiners would signal to listeners whether to predict the suffix-triggered shortening in plural *laps* or *badges*, or its absence in singular *lap* and *badge*. By contrast, no such interaction between Match and Context should be present when distinguishing *lap/lapse* or *badge/badger*, because both nouns in each pair are singular, and so cannot interact with morphosyntax in this way.

The second prediction we can make is that – for duration-related cues, at least – any Match effect should be smaller for ancillary morphophonetic cues than direct phonetic cues. This was observed in the current study, and in Cohen (2024), but since the two cue types arose from a different process, the comparison is confounded. However, evidence from production also supports this prediction. Sugahara and Turk (2009) report lengthening preceding morpheme boundaries, relative to segmentally equivalent monomorphemic sequences. Thus, speakers lengthened the rhyme of *puff* in *puffing* more than in *puffin*. This lengthening before morpheme boundaries could serve to counteract the shortening caused by suffixation. As a result, listeners may simply not learn ancillary morphophonetic cues as well, or may not rely on them as much as direct phonetic cues. What is more, if direct phonetic cues can distinguish different lexemes, while ancillary morphophonetic cues distinguish different forms of the same lexeme, then listeners may also disregard the latter simply because getting them wrong is less likely to cause confusion.

To our knowledge, no such study of this sort has been conducted. We believe the methods of the experiments presented here could be easily adapted to run such a study.

## Conclusion

The two experiments reported here suggest that listeners make use of both direct phonetic cues and ancillary morphophonetic cues to aid their real-time speech processing in both English and Spanish. The links between morphosyntactic context and inflectional forms also affect how listeners use ancillary morphophonetic cues: When the morphosyntactic context allows listeners to predict upcoming morphological forms, they can make better use of those cues, and especially so in Spanish, whose richer morphosyntax reinforces the phonetic information. Listeners are resourceful creatures: they let no cue go unexploited.

## Footnotes

1 The stress marks are included as an aid to the reader, and are not part of Spanish orthography for these words.

2 The metalinguistic mention context is awkward in English, especially given that *the* is an easily available alternative that preserves every other aspect of the sentence’s structure, making comparison across Agreeing and Non-Agreeing contexts straightforward. However, Spanish does not offer such an option, so the metalinguistic mention construction was the only way to present target nouns in a context lacking any agreeing determiner. Since we wanted to make direct comparisons between Spanish and English, we elected to use the metalinguistic mention context in English as well as Spanish.

3 Adding sentence context did not improve the model fit. This might have been because it was almost perfectly collinear with duration: As Table 1 indicates, stems from Non-agreeing sentences (bottom row) were substantially longer than their counterparts from Agreeing sentences (top row).
